# Biobased Nanomaterials—The Role of Interfacial Interactions for Advanced Materials

**DOI:** 10.1021/acs.chemrev.2c00492

**Published:** 2023-01-31

**Authors:** Monika Österberg, K. Alexander Henn, Muhammad Farooq, Juan José Valle-Delgado

**Affiliations:** Department of Bioproducts and Biosystems, School of Chemical Engineering, Aalto University, Vuorimiehentie 1, 02150Espoo, Finland

## Abstract

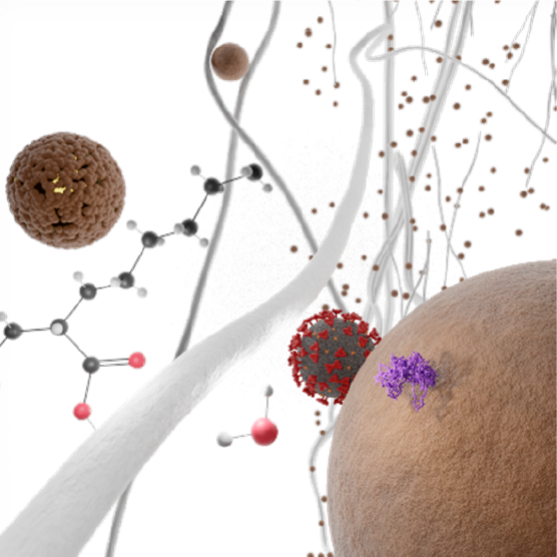

This review presents recent advances regarding biomass-based nanomaterials, focusing on their surface interactions. Plant biomass-based nanoparticles, like nanocellulose and lignin from industry side streams, hold great potential for the development of lightweight, functional, biodegradable, or recyclable material solutions for a sustainable circular bioeconomy. However, to obtain optimal properties of the nanoparticles and materials made thereof, it is crucial to control the interactions both during particle production and in applications. Herein we focus on the current understanding of these interactions. Solvent interactions during particle formation and production, as well as interactions with water, polymers, cells and other components in applications, are addressed. We concentrate on cellulose and lignin nanomaterials and their combination. We demonstrate how the surface chemistry of the nanomaterials affects these interactions and how excellent performance is only achieved when the interactions are controlled. We furthermore introduce suitable methods for probing interactions with nanomaterials, describe their advantages and challenges, and introduce some less commonly used methods and discuss their possible applications to gain a deeper understanding of the interfacial chemistry of biobased nanomaterials. Finally, some gaps in current understanding and interesting emerging research lines are identified.

## 1 Introduction

Worldwide population growth, combined with widespread increases in energy and materials use, contributes significantly to global warming, pollution, and diminution of Earth’s natural resources. To maintain the current standard of living while protecting the environment, there is a demand for a materials paradigm shift to a circular materials bioeconomy. This includes more efficient recycling, upcycling and sustainable use of renewable resources. In the transition from fossil-based resources to renewables, biobased nanomaterials are pursued as one of the most promising alternatives to address these challenges. Cellulose nanomaterials (CNMs) are by far the most researched plant-based nanomaterial,^1−4^ followed by lignin nanoparticles (LNPs).^5,6^ Other renewable nanomaterials include chitin and chitosan,^7−9^ starch,^10,11^ and hemicelluloses,^12−14^ but these have, to date, attracted less attention. Biobased nanomaterials combine the possibilities of nanotechnology with the typical advantages of renewables, like abundance, biodegradability, recyclability, biocompatibility, and low production costs. Harnessing their unique inherent properties, advanced materials that not only replace but outperform the current synthetic materials can be developed from biobased nanomaterials.

### 1.1 Sources for Biobased Nanomaterials

The main biopolymers in nature with the ability to form nanomaterials are polysaccharides, polyphenols, and proteins. Polyphenols are abundantly found in plants, while proteins are mainly synthesized by animals or bacteria. Polysaccharides are widely present in any living material. Wood and vascular plants contain the polysaccharides cellulose and hemicellulose, and lignin, a complex polyphenolic polymer. Wood and plant fibers have been used for centuries in materials like paper and textiles, and plant-based biorefineries have been optimized for high yields at low cost and minimal environmental burden. While there is still high demand for macroscopic pulp fibers for packaging and tissue, and polymeric cellulose for textiles and bioenergy, existing biorefineries are also excellent sources of biomass for CNM and lignin nanomaterial (LNM) production. A sustainably managed forest reduces soil degradation, acts as a carbon sink, has a positive impact on biodiversity, can create income, and provides food and recreation for people.^15−17^ At the same time, it is an abundant source of timber and fibers. Virgin wood fibers have been used extensively for CNM production with excellent results, but in the interest of efficient use of resources, other feedstocks should be considered. Agriculture waste residues are also common feedstocks for both CNMs and LNMs with the benefits of having more frequent harvests. Lignin is available as a side stream from the pulp and paper industry and biorefineries, but it is currently mainly burned for energy. Efficient utilization of the lignin in materials would boost the transition to more energy-efficient processes and greener energy sources, and lead to a positive carbon handprint by binding the carbon in products for a longer time.^18,19^

CNMs can also be obtained from nonplant sources like algae, tunicates, and different bacteria species.^20−22^ In particular, bacterial cellulose has been intensively studied in the last two decades, with a special focus on biomedical applications.^23^ Nevertheless, the isolation and preparation of nonplant CNMs still need optimization for large-scale production.

Sources for other natural nanomaterials include, for example, crustacean shell waste and fungi for extraction of chitin nanofibrils, or biotechnical means for the controlled preparation of silk nanofibrils.^24,25^ Their extraction is still more energy intensive (chitin nanofibrils from crustaceans) or available at a smaller scale (silk nanofibrils or chitin from fungi) than the plant-based nanomaterials.^26^ Therefore, we are focused on nanomaterials derived from plants, and hence the plant cell wall structure is reviewed next.

### 1.2 The Plant Cell Wall

Plant cell walls are complex, dynamic structures with multiple roles, including providing strength, expandability, modularity, and a barrier against pathogens.^27^ In nature, elementary constituents range from oligosaccharides and polysaccharides to lignin and fibers found in biomass. Furthermore, they are multifunctional and stereoregular and show a wide variety of complex structures based on small chemical variations. Understanding both the hierarchical structures and function of these constituents in nature is important for the efficient design of functional biobased nanomaterials, hence these are briefly reviewed in this section.

Typically, the plant cell is constituted of primary and secondary cell walls, and the cells are bound together by the middle lamella. The cell wall consists of cellulose, hemicelluloses (xylan, glucuronoxylan, xyloglucan, arabinoxylan, mixed linkage glucan, or glucomannan), lignin, and pectic polysaccharides. The middle lamella is mainly made of lignin and pectin. The main polymer in the cell wall is cellulose. Cellulose is a polysaccharide consisting of chains of β-(1–4)-linked-d-glucose repeating units. These chains assemble into bundles, called microfibrils, held together by hydrogen bonds and van der Waals (vdW) interactions.^28^ The width of the microfibrils depends on their biological origin, ranging from approximately 3–4 nm for trees to 20 nm for algae. While the smallest fibrils were long thought to consist of 36 cellulose chains, molecular dynamics simulations recently suggest that the smallest microfibrils consist of only 18 cellulose chains.^29−31^

The crystalline structure of the cellulose microfibrils is another aspect of the cell wall structure that recent advances in measurement methods have been able to shed light on. The measured crystallinity of native cellulose is usually in the range of 50–80%, which has led to the traditional assumption that the microfibril consists of crystalline domains interrupted by amorphous domains along the length of the fibril. However, recent neutron scattering studies have shown that these unordered domains are very short, only 1–2 nm, and should be called defects or disordered regions instead and the crystallinity is in fact much higher.^32^

Aligned microfibrils form thin discrete layers with randomly changing fibril orientation when traversing through the primary cell wall. The microfibrils are surrounded by hemicelluloses. The hemicelluloses are branched polysaccharides with a backbone consisting of neutral sugar units, while the branches may be neutral or negatively charged. The hemicelluloses are bound to the cellulose fibrils via hydrogen bonds and vdW attraction. It has been suggested that the hemicelluloses facilitate cell wall expansion by preventing the close packing of cellulose fibrils and thus weakening the mechanical strength of the cell wall.^27^ Measurements of the total sugar composition of cell walls from different tissue of *Arabidopsis thaliana* has revealed that not only the cell wall composition vary between different plants, but every tissue type has a different polysaccharide composition.^33^

Polyphenolic lignin is found in the secondary cell wall and is essential for the structural integrity of the cell wall and the stiffness and strength of the stem and root. The monomeric precursors for lignin are *p*-coumaryl, coniferyl, and sinapyl alcohols. Polymeric lignin is composed of an integrated network of aromatic units derived from the radical coupling of these monomers. The basic units are called *p*-hydroxyphenyl, guacil, and syringyl, denoted as H, G, and S, respectively. They differ in the level of methoxylation of the aromatic ring: H-lignin being non-methoxylated, G-lignin containing one methoxy group, and S-lignin having two methoxy groups.

Lignin typically comprises 20–30% of the lignocellulosic biomass, however, the exact structure of the complex polymer varies greatly depending on the botanic origin of the lignin and the isolation process. Hardwood lignins contain a similar amount of G- and S-lignins, while softwood lignins contain more G-units. Herbaceous lignins contain all three units. Biorefineries are optimized for high yield of the polysaccharides, which leads to severe changes in the lignin structure. For a detailed understanding of the chemical structure of technical lignins, the reader is referred to recent papers,^34−37^ here we give a very general overview.

To remove lignin from the biomass, the ester and ether bonds in native lignin are cleaved, breaking the lignin into smaller fragments of different chemical structures.^37^ However, radical coupling can lead to the formation of new carbon–carbon bonds and condensation into less soluble lignin.^34^ Some processes also introduce new functional groups, such as the introduction of sulfur during the Kraft or sulfite processes. In general, lignin degradation during various technical processing results in a decrease in aliphatic OH groups, β-O-4 linkages, and total oxygenated aliphatic moieties. In contrast, the amount of phenolic OH and saturated aliphatic moieties increase.^37^ Due to these various reactions, technical lignins are complex mixtures of molecules with varying molecular weight and chemical structure, and detailed nuclear magnetic resonance (NMR) spectroscopic analysis has identified several hundreds of different signals.^37^

### 1.3 Scope and Goals of the Review

There are numerous reviews on the production, properties, and applications of both CNMs^2,38−40^ and LNPs,^5,6^ hence these aspects are only briefly discussed here. In contrast, their interfacial interactions have surprisingly garnered less focus even though the properties of nanomaterials are governed by their surface properties. This review aims at describing the specific surface properties of plant-based nanomaterials and how the surface properties affect their interactions with solvents, polymers, proteins, and other compounds relevant to their performance in applications. Although our discussion is centered on CNMs and LNPs, some model thin films from regenerated cellulose or lignin are also mentioned to highlight how differences in surface chemistry and morphology affect the material properties and the interactions with other molecules. We expect that this information will enable efficient choice of the most suitable nanoparticles for specific applications and pave the way for the development of new innovative materials solutions. We furthermore hope that this review will elucidate the potential of surface-sensitive techniques for understanding the behavior of plant-based nanomaterials and inspire more scientists to explore these methods. We focus on the lignocellulosic nanomaterials, so other natural nanoparticles will not be reviewed. Material applications of silk nanofibers is an emerging field that holds great future potential, and we refer the interested reader to some recent papers on the topic.^41−44^ Starch nanoparticles have attracted interest for their ability to encapsulate, protect, and orally deliver bioactive components because of their diverse functionality, high biocompatibility, and environmental friendliness.^45^ Their production and application are reviewed in more detail by Qiu et al.^45^ and Kim et al.^46^ Chitin and chitosan nanoparticles have been actively explored in biomedical applications.^47,48^

To facilitate a more thorough discussion on nanoparticle interactions, the basics of intermolecular and surface forces are recapped in section 2. In section 3, cellulose nanomaterials are discussed, first introducing the main points regarding their surface properties that will affect their interactions, then discussing their interactions in various media and finally with polymers, proteins, and cells. Section 4 is devoted to lignin nanomaterials. Because intermolecular interactions play a decisive role in the supramolecular assembly of LNPs, these are first discussed in detail before reviewing the interactions of LNPs with media and other substances. The combination of lignin and cellulose in nanomaterials is discussed in section 5. Our current understanding of the interfacial interactions of nanomaterials is based on the large variety of analysis methods that have been applied. In section 6, these methods are briefly described including their advantages, drawbacks, and especially what information they provide. In the final section, we discuss what main conclusions can be drawn based on the current literature and what are still open questions and possible emerging fields.

## 2 Intermolecular and Surface Interactions

In this section, some common intermolecular and surface forces are briefly introduced. We focus on forces that are relevant for the systems discussed in this review, such as vdW, electrical double layer (EDL), and hydration forces, as well as interactions induced by adsorbed polymers. For a more comprehensive description, the reader is referred to the textbook by Israelachvili.^49^

### 2.1 DLVO Forces

**Figure 1:**
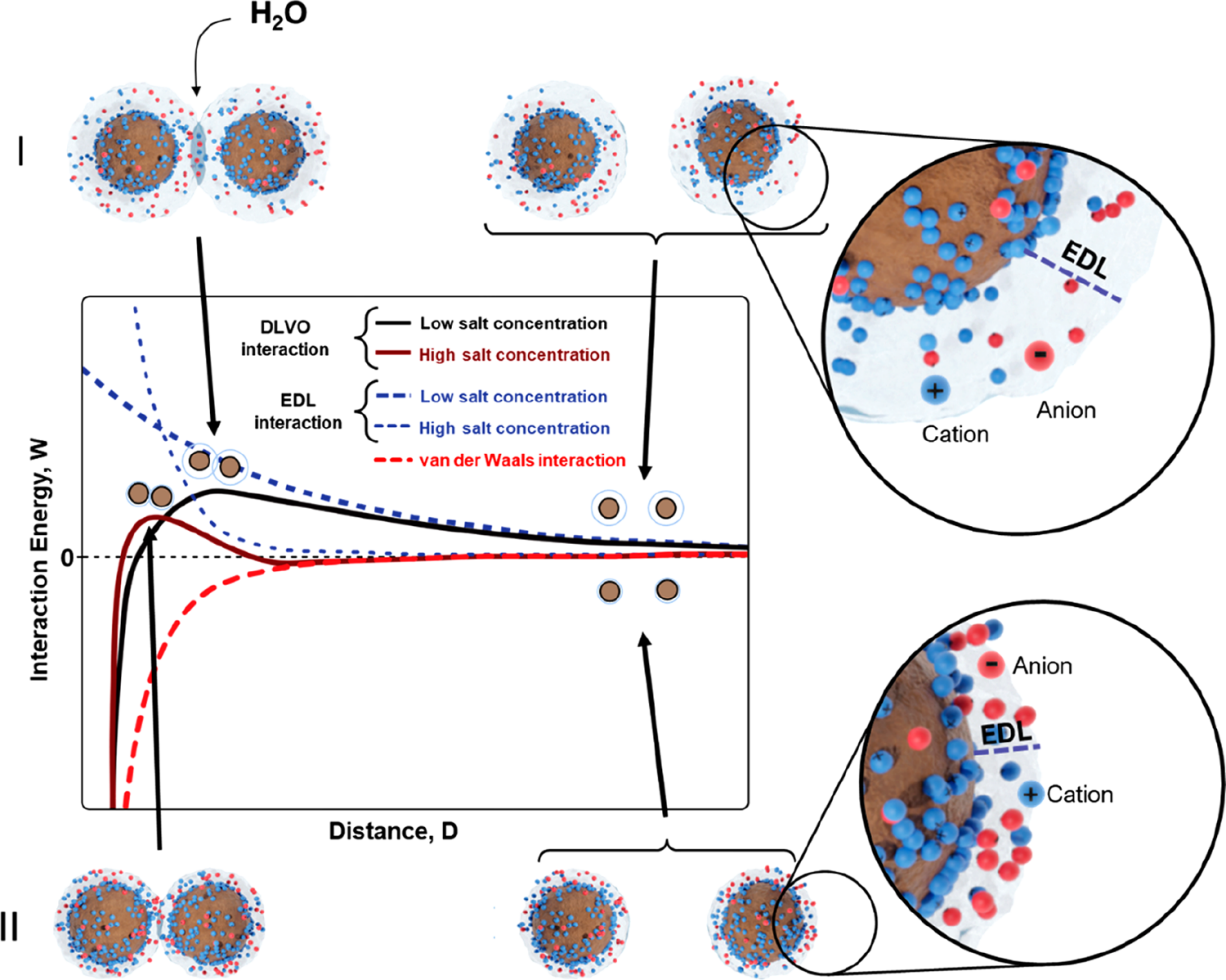
Schematic of interaction energy–distance profiles between two negatively charged particles, focusing on the effect of the ionic strength on the DLVO interaction (solid curves). The interaction energy (and consequently the force) is zero when particles are far apart, but when they approach each other, the overlap of their EDLs generates a repulsion of osmotic nature (water flows in between the particles to dilute the accumulation of ions in the overlapped EDLs). At low salt concentrations, the EDL is thick, and the EDL repulsion starts at a larger separation between the particles (I). On the contrary, at high salt concentrations, the EDL is thinner, which allows particles to come closer to one other before the EDL repulsion arises (II). When the salt concentration is high enough, the attractive van der Waals forces can overcome the repulsive EDL force, which leads to particle aggregation.

The colloidal stability of nanomaterials can be discussed in the framework of the classical DLVO theory,^50,51^ named after Derjaguin, Landau, Verwey, and Overbeek. This theory suggests that the interaction between two particles across a liquid at any distance equals the sum of the EDL force and the vdW interactions. The DLVO theory is often a good first estimate for interparticle forces at separations down to about 5 nm. A qualitative overview of the DLVO forces as a function of particle separation is shown in Figure 1.

The vdW forces originate from the correlation between permanent or induced electric dipoles of molecules approaching one another. The vdW interaction energy (*W*_vdW_) between two molecules decays quickly with separation (eq 1).
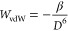
1where the molecular properties like dipole moments or polarizabilities are included in β, and *D* denotes the distance between the molecules. Because even nonpolar molecules can have induced dipole moments, we note that the vdW forces are present between all molecules and surfaces. The vdW interaction energy between particles or surfaces is the sum of the vdW interaction energies between all of their constituting molecules, and it depends on both the geometry and molecular properties of the system. The molecular properties are included in the Hamaker constant *A*_H_, which, according to the Lifshitz theory, can be connected to the dielectric permittivity ε and refractive index *n* of the interacting particles or surfaces and the medium in which they interact. Thus, the nonretarded *A*_H_ for particle 1 interacting with particle 3 across medium 2 can be expressed by eq 2:
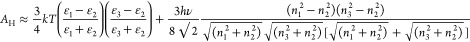
2where *k* is the Boltzmann constant, *T* is the temperature, *h* is Planck’s constant, and ν is UV absorption frequency. The expression for interaction energy *W*_vdW_ between a spherical particle of radius *R* and a flat surface, assuming that *D* ≪ *R*, is given by eq 3:
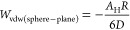
3Considering that force *F* and interaction energy *W* are related by the equation , the corresponding van der Waals force is given by eq 4.
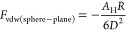
4The vdW interaction energy and forces between two spherical particles of radius *R*_1_ and *R*_2_ are given by eqs 5 and 6, respectively.
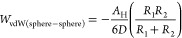
5
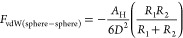
6From these equations, we note that the vdW forces decay much slower between particles than between molecules, affecting the overall interactions at separations up to a few nm. It can be seen in eqs 2–6 that the vdW interaction is always attractive (*W*_vdW_ < 0; *F*_vdW_ < 0) between particles of the same nature, it is stronger in the air or nonpolar media than in water, and it is strong for polar particles.

**Figure 2:**
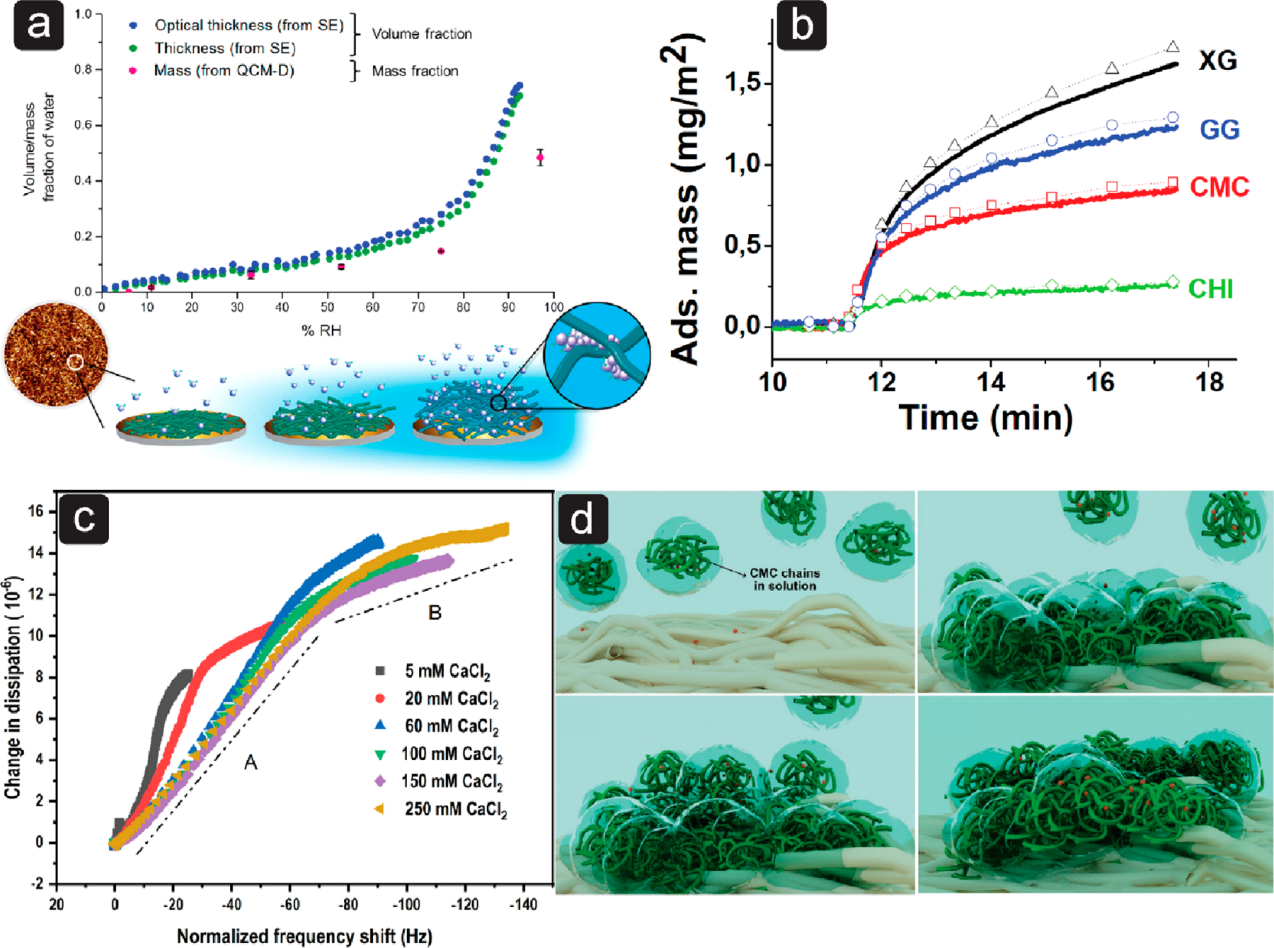
(a) Thickness, optical thickness, and mass fractions of water in TEMPO–CNF thin films as a result of water vapor uptake as a function of relative humidity and schematic illustration of the water vapor uptake of TEMPO–CNF thin films at different humidity levels.^88^ Reproduced with permission from ref (88). Copyright 2017 American Chemical Society. (b) Adsorbed mass as a function of time for anionic (carboxymethyl cellulose, CMC), nonionic (xyloglucan, XG; guar gum galactomannan, GG), and cationic polysaccharides (chitosan, CHI) on CNF.^89^ Reproduced from ref (89), used under open access from BioResources. (c) Change in dissipation as a function of normalized frequency shift during the adsorption of CMC on CNF model surfaces in aqueous CaCl_2_ environments.^90^ (d) Schematic of CMC adsorption in the presence of CaCl_2_. (c,d) Reproduced with permission from ref (90) under Creative Commons CC-BY license. Copyright 2017 American Chemical Society.

For charged particles or surfaces in aqueous media, we also have to take into account the contribution of the EDL force, which often dominates the interaction at long distances. The EDL force arises from the overlapping of the EDLs formed around the charged particles, and its magnitude depends on the charge of the particles (the higher the charge, the stronger the EDL repulsion), whereas the range of the repulsion or thickness of the EDL is described by the Debye length κ^–1^, which correlates inversely with the ionic strength *I* = (∑_*i*_*c*_*i*0_*z*_*i*_^2^)/2 of the aqueous medium as described by eq 7:
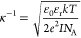
7where *c*_*i*0_ is the concentration of ions *i* with valence *z*_*i*_ in the bulk, *N*_A_ is Avogadro’s number, *ε*_0_ is the dielectric constant of vacuum, ε_r_ is the relative permittivity of the medium, and *e* is the elementary charge. For particles with similar charge, the EDL force is repulsive, while oppositely charged particles have attractive EDL force.

Different mathematical expressions can be obtained for the EDL interaction energy and force depending on the conditions of the interaction. In the simplest case, the EDL repulsion decays exponentially with the separation between the particles (eqs 8 and 9):
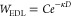
8
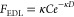
9where parameters like the surface potential and radius of the particles, the temperature, and the relative permittivity of the medium are included in factor *C*.

DLVO theory is often a good first approximation when predicting the stability of biobased nanoparticle dispersions, and a few main conclusions can be made already here, while specific cases are discussed in more detail below (see sections 3.1 and 4.3). A high charge of the particles results in strong EDL repulsion, whereas increasing the ionic strength in the medium leads to shorter *κ*^–1^, that is, faster decay of the EDL repulsion. The screening of the EDL repulsion by increasing the ionic strength enables the particles to come close enough for the attractive vdW forces to dominate, leading to aggregation.

### 2.2 Non-DLVO Forces

Not all systems are well described by the DLVO theory. Hydrophilic surfaces may strongly bind water molecules, leading to a repulsion at short distances, compensating the attractive vdW interactions and enabling colloidal stability even in conditions where aggregation is expected. Hydration forces depend on the ionic species in the media, their concentration, as well as surface roughness.^52^ Usually it is monotonically repulsive, but oscillatory hydration forces are also possible.^53−55^ In the presence of polymers, both attractive and repulsive forces that cannot be described by DLVO theory may occur. Very common for plant-based nanomaterials is the presence of steric repulsion. This repulsion is generally present when surfaces are fully covered with polymers that have good solubility in the media and are hence adsorbed in an extended conformation, but it can also be due to the roughness of the model substrates used in the studies. When two such polymer-coated surfaces approach, the polymers need to acquire a new, more collapsed, and energetically less favorable conformation, leading to repulsion. EDL repulsion sometimes enhances the steric repulsion, leading to a more long-ranged repulsion than for pure EDL. This force is often called *electrosteric repulsion*. Hence the range of steric repulsion may vary between some subnanometers to hundreds of nanometers. If the adsorbed amount of polymer is low and the particles are not fully covered, bridging attraction may be observed. This occurs when a polymer chain adsorbed to one surface or particle is attracted to another particle. The bridging attraction can range to several nm. For a more thorough review on the interactions between surfaces covered with polymers or polyelectrolytes, the reader is referred to the report by Claesson et al.^56^

The above-mentioned forces are important for colloidal stability in nanoparticle dispersions and adsorption of polymers and proteins because they range over several nm. Intermolecular forces like hydrogen bonds, π–π interactions, Lewis acid/base interactions, and interactions between ions and dipoles play a role in the solubility of polymers and rigidity and mechanical properties of supramolecular assemblies like the CNF, LNPs, or mechanical properties of composites. However, one needs to keep in mind that the range of these interactions is less than 0.5 nm. Hence particles or molecules first need to come very close to one another before they become relevant.

## 3 Cellulose Nanomaterials

A nanomaterial is defined as a material with any external dimension in the nanoscale (between 1 and 100 nm) or having internal or surface structure in the nanoscale.^57^ Nanocellulose is the general term for cellulosic nanomaterials (CNMs), which include cellulose nanocrystals (CNCs), cellulose nanofibrils (CNFs), and bacterial cellulose or bacterial nanocellulose (BNC). CNCs are sometimes also called cellulose whiskers or nanocrystalline cellulose, while especially in older literature, microfibrillar cellulose or nanofibrillar/nanofibrillated cellulose are used as synonyms for CNF. In 2011, standard nomenclature for CNMs was suggested, and now the generally used abbreviations are CNC and CNF.^58^ The morphology and surface properties of these depend on the cellulosic feedstock and production method. A wide range of methods can be combined for a thorough characterization of CNMs, these include electron microscopy and atomic force microscopy (AFM) to determine size and morphology, X-ray diffraction methods for degree of crystallinity, conductometric titration for surface charge, zeta potential measurements, and dynamic light scattering for colloidal stability, among others. Rheology measurements give information on viscoelasticity of hydrogels, and spectroscopy can be used to determine chemical composition, including surface sensitive X-ray photoelectron spectroscopy and Fourier transform infrared and nuclear magnetic resonance spectroscopy for bulk analysis. In this review, we focus on methods used to study interactions of CNMs, including quartz crystal microbalance with dissipation monitoring (QCM-D), surface plasmon resonance (SPR), atomic force microscopy (AFM) force spectroscopy, and calorimetry.

CNCs are mostly produced via acid hydrolysis using sulfuric acid or selective oxidation of the cellulose primary hydroxyl groups using 2,2,6,6-tetramethylpiperidine-1-oxyl (TEMPO) mediated oxidation, leading to highly crystalline whiskers with either sulfate ester groups (−OSO_3_^–^) or carboxylic groups (−COO^–^) at the surface, respectively. The sulfate ester group is a strong acid, hence CNCs produced via sulfuric acid treatment are negatively charged irrespective of the pH. Sulfate content ranging from 200 to 330 mmol/kg of CNCs and zeta potentials from −35 to −45 mV have typically been reported.^59,60^ The carboxylic groups, on the other hand, are weak acids and protonated at low pH and deprotonated at higher pH, making the charge of TEMPO CNCs pH-dependent. The p*K*_a_ value of acetic acid is 4.7, but because dissociation of charged groups is impeded by other charged groups in the vicinity, the effective p*K*_a_ for the carboxylic groups on CNCs is slightly higher. Degrees of oxidation up to 0.1 have been reported.^61^ The CNCs can be produced via hydrolysis using hydrochloric acid, but these CNCs are, in practice, uncharged and hence have very low colloidal stability.^62^ Cationic CNCs can be produced by reacting the sulfate ester containing CNCs with 2,3-epoxypropyl trimethylammonium chloride (EPTMAC) resulting in a zeta potential of +30 mV. Recently an alternative method using reactive eutectic media to produce cationic CNCs was introduced.^63^ For a more thorough assessment of CNC production, chemistry, and applications, the reader is referred to a comprehensive review by Habibi et al.^2^

CNFs are produced by mechanical disintegration of cellulose pulp, sometimes aided with enzymes. Using bleached pulp as a starting material results in fibrils with a low negative charge, consisting of cellulose and hemicelluloses, with zeta potential around −3 mV at pH 8. The hemicellulose content on the fibril surface is very difficult to experimentally determine but may still affect the interactions, for example, by introducing some charged groups and by adding amorphous structures on the CNF surface. The pulp can also be chemically modified prior to disintegration to introduce a higher charge and facilitate fibrillation using less energy. The two most common approaches are TEMPO-mediated fibrillation and carboxymethylation, which both introduce anionic groups to the fibrils.^64,65^ Cationic CNF can be produced via reacting the pulp with EPTMAC or periodate and Girard’s reagent T prior to disintegration.^66,67^ The sign and magnitude of the surface charge affect the interaction of CNF in applications, hence these factors are important to consider. Charge densities of 0.5 and 0.9 mequiv/g have been reported for carboxymethylated and TEMPO-oxidized pulp used for the production of highly anionic CNF.^65,68^ This resulted in a zeta potential at pH 8 of −39 mV for 0.9 mequiv/g charged TEMPO-oxidized pulp. Just as for CNCs with COOH groups, the charge is pH-dependent for these CNFs.

The surfaces of CNMs provide hydroxyl groups, and for the more anionic variants, also carboxylic groups that are accessible for chemical modification. To combine the advantages of CNMs with the controllability of synthetic chemistry, extensive research has been devoted to tailoring the CNMs for various applications via chemical modification. The challenges concerning accessible surface area, if the colloidal stability is not considered during modification, are discussed in sections 3.1 and 3.2, but for a more comprehensive review of the available types of modified CNMs, we refer the reader to recent reviews.^38,69^ In the future, green chemistry concepts like chemoenzymatic surface modification^70^ need to be considered. The toxicity and degradability of CNMs are affected by chemical modification,^70,71^ and we envision that this will lead to an increased focus on alternative and milder approaches to the tailoring of the surface chemistry of CNMs. One approach in this direction is the use of lignin-containing nanocellulosic materials which are briefly reviewed in section 5.

### 3.1 Interaction of CNMs with Water and Ionic Solutes

Water interacts with cellulose both at molecular and supramolecular scales, and such water–cellulose interactions are commonly present in Nature (e.g., in wood). Understanding and tailoring those interactions can lead to new, advanced applications of cellulose-based materials.^72^ Fundamental studies on CNM dispersions using calorimetry, rheology and scattering techniques, and on CNM thin films using surface-sensitive methods like QCM-D, SPR, and AFM with modeling tools, have increased our understanding of how CNMs interact with water and are affected by ionic strength and pH. The main findings from these studies and their implications on applications are addressed here.

Cellulose can form hydrogen bonds with water molecules through the abundant hydroxyl groups present in the cellulose molecular structure. Due to their larger surface area, there are considerably more accessible hydroxyl groups on the surface of CNFs than on macroscopic fibers, which explains the larger hydration state of cellulose at the nanoscale. Nevertheless, water does not dissolve cellulose. The formation of multiple intra- and intermolecular hydrogen bonds favors the formation of well-packed crystalline assemblies of the cellulose molecules within the nanofibrils that do not dissolve in water. Hydrophobic interactions, due to the amphiphilic nature of cellulose, also contribute to its insolubility in water.^73,74^ Furthermore, it is estimated that the dissolution of cellulose nanofibrils in water is not entropically favorable. This is because the partial increase in the mobility of the cellulose molecules upon dissolution is overcome by a decrease in the configurational freedom of a larger number of water molecules that are hydrogen-bonded to the cellulose molecules.^73,75^

Although insoluble in water, cellulose nanomaterials swell in the presence of water. Water molecules cannot penetrate the crystalline nanocellulose assemblies, but they can access the less ordered amorphous regions and the space between nanocellulose building blocks in 2D and 3D networks. QCM-D experiments have revealed that the swelling of cellulosic thin films is both governed by the degree of crystallinity of the cellulose materials and by the morphology and porosity of the films. Thus, comparing cellulose films with different crystallinity, Aulin et al. observed significant swelling of CNC films.^76^ Because the tightly packed CNCs are not expected to swell, the observed swelling can be explained by the adsorption and accumulation of water molecules on the surface and in the spaces between the nanocrystals, further increasing the separation between them. In the same line, water vapor adsorption experiments carried out by Tammelin et al., also using the QCM-D technique, showed that a cellulose film with a degree of crystallinity of about 60% swelled more than a chemically identical but highly amorphous film when the relative humidity was 97%.^77^ The reason for this at-first-unexpected result is the nanoscale porosity and, consequently, the larger surface area and higher number of hydroxyl groups accessible for water molecules in the more crystalline film. The importance of the film structure for water adsorption was also pointed out by Reishofer et al., who observed that both the preparation method and the applied treatment (e.g., drying at elevated temperature) affected the water uptake of highly amorphous cellulose thin films, especially at high relative humidities.^78^ Similarly Niinivaara et al. observed that the ratio between crystalline and amorphous regions was not the only factor determining the swelling of 2D films where CNC and amorphous cellulose were combined to mimic plant cell walls.^79^ In this system, the total interfacial area between CNC and amorphous cellulose was also suggested to play a role in swelling.

EDL forces also govern the swelling behavior of films and hydrogels made of charged CNMs. Hence charge density of the CNMs, ionic strength, and polarity of the media will play a decisive role in the behavior of CNMs in aqueous media, as has been shown in early studies. Ahola et al. studied the effect of the surface charge on swelling and interactions of CNF model films using QCM-D and AFM measurements.^80^ They observed a larger swelling in the water of the highly charged (carboxymethylated) CNF compared to the low charged (noncarboxymethylated) counterpart. The repulsion between charged nanofibrils and osmotic effects (Donnan equilibrium) can explain the larger swelling of highly charged CNF. Increasing the salt concentration decreased the swelling of the films because the higher number of ions in the medium screened the repulsion between charged groups and reduced the osmotic pressure difference between the inside and outside of the film. In addition, an increase in film swelling was also observed, more remarkable in the case of carboxymethylated CNF, when increasing the pH from 3.5 to 10, in line with an increase in surface charged groups due to the deprotonation of carboxyl groups. The film swelling detected by QCM-D correlated with the surface forces measured between a cellulose microsphere and the CNF model films using the AFM colloidal probe technique. The observed repulsive forces were of longer range for highly charged CNF, and they changed with the salt concentration and the pH, in agreement with the swelling state of the film. The repulsive force was of longer range than expected for pure EDL repulsion, and it was assumed that steric repulsion between the swollen layers was also present.

Interesting effects of ionic solutes on CNF hydrogels and adsorption of negatively charged polymers onto cellulosic substrates have recently led to a renewed interest in the interactions between ions and cellulose, suggesting that DLVO theory is not enough to describe their behavior. Arola et al. used a combination of small deformation oscillatory rheology and molecular modeling to gain an understanding of the effect of salt on rheological properties of CNF hydrogels.^81^ They found that already at ion concentrations of 1 mM, various monovalent sodium salts caused crowding of hydrogels and subsequently argue that screening of the EDL repulsion could not explain this phenomenon. Instead, they suggest that the water molecules become more ordered, leading to a stronger hydration layer. This correlates with the results by Ahola et al., who found no deswelling of low-charged native CNF films at 1 mM NaCl and no change in the repulsive force upon approach. However, higher concentrations (10 or 100 mM) of electrolyte resulted in slight deswelling of CNF films, and the water-binding was pH-sensitive, suggesting that, even for very low charged CNF, electrostatics also play a role although the effects were much more pronounced for highly charged CNF.^80^

Divalent cations, especially Ca^2+^, are known to be able to form ionic cross-links between carboxylate groups and hence stabilize hydrogels made from either TEMPO-oxidized cellulose nanofibers (TOCNF) or a mixture of alginate and CNF.^82^ Recently, Ju et al.^83^ investigated the coordination complexes between various metal ions and carboxylated CNF and showed that the cross-linking density increases in the order Zn^2+^ < Ca^2+^ < Cu^2+^ < Al^3+^. The interaction between metal ions and CNF was visualized by a shift of the Fourier transform infrared spectroscopy (FTIR) peaks to higher wave numbers for bands associated with the carboxylate group. Ca^2+^ ions are also efficient coagulants for filaments prepared via wet spinning of TOCNF.^84^

Lombardo et al.^85^ used isothermal titration calorimetry to reveal the interactions between divalent cations and CNCs. They showed that the interaction was endothermic and driven by the increase in entropy upon adsorption of ions due to an increase in the degree of freedom for released water molecules. This entropy gain compensated for the unfavorable endothermic enthalpy. A comparison of CNCs with sulfate or carboxylate groups showed that the nature of the ionizable group on the CNC affected the pH dependence of the interactions. The adsorption of cations to CNCs with carboxylic groups was clearly pH-dependent, showing that carboxylic groups needed to be deprotonated, while sulfate groups were less sensitive to the pH. They concluded that the adsorption of ions of the same net charge followed the same mechanism.

As already mentioned, the abundant hydroxyl groups on cellulose surfaces are responsible for the adsorption of water molecules. CNMs are especially hygroscopic due to their larger surface area. Combining QCM-D and spectroscopic ellipsometry in a water vapor adsorption study, Niinivaara et al. concluded that a 1 nm thick layer of water molecules was strongly adsorbed on the surface of the individual CNCs in the film,^86^ in excellent agreement with the results from Reid et al. on the swelling of CNCs studied by SPR.^87^ In a similar work, Hakalahti et al. distinguished between three different stages for the adsorption of water vapor on TEMPO–CNF at different relative humidity (RH) values that were well fitted with a Langmuir/Flory–Huggins clustering model. These three stages were the specific adsorption of water molecules at low RH (below 10%), a buildup of water multilayer at intermediate RH (10–75%), and clustering of water molecules at high RH (above 75%) (Figure 2a).^88^

The water molecules strongly bound to cellulose surfaces have different properties from the bulk water. They cannot freeze due to conformational restrictions; thus this is called *nonfreezing water*. In contrast, the term *freezing water* is used to describe the weakly bound water molecules confined in the pores of nanocellulose networks, with a shifted temperature for solid–liquid transition compared to bulk water. Different experimental techniques (nuclear magnetic resonance or NMR, neutron scattering, differential scanning calorimetry or DSC) and molecular dynamics simulations have confirmed the presence of freezing and nonfreezing water in cellulose fibrillar materials.^91−96^ The properties of these confined water species have been exploited in DSC-based thermoporometry to quantify the porosity of cellulose materials.^91,94,95^

The hygroscopicity of cellulose has very often been seen as a negative property because the integrity and mechanical properties of cellulosic materials (paper, cardboard, composites, etc.) are usually dramatically decreased in the presence of water. To prevent the adsorption of water and degradation of mechanical properties in wet or humid conditions, different approaches have been applied for the hydrophobization of cellulose surfaces to expand the utilization of cellulose to, for example, barrier and packaging materials. Those approaches involve changes in surface chemistry of CNF via the adsorption or covalent attachment of hydrophobic molecules,^97^ nevertheless, hydrophobized CNFs do not form strong films or nanopapers because the amount of interfibrillar hydrogen bonds responsible for the strength of cellulose networks is severely decreased. A cleverer approach to avoid altering the mechanical properties of the final product is to form the cellulose network first and then hydrophobize its exposed surfaces via vapor deposition, covalent attachment, or simply adsorption of hydrophobic molecules, polymers or nanoparticles, which could be combined with treatments to enhance the roughness of the material at nano- and microscale.^98−101^ Similarly, it has been shown that CNCs with an increasing lignin content does not result in barrier materials with low water vapor transmission rates because the presence of the hydrophobic lignin makes the materials more porous.^102^ Nevertheless, it has also been shown that the strength of cellulose fibers and nanocellulose films is increased to some extent by humidity.^103−106^ Surface-bound water can increase strength by mediating hydrogen bonds between hydrophilic structures that would otherwise be too far away from each other to form hydrogen bonds. This ultimately increases the hydrophilic structures’ reach to interact with one other and therefore increases the number of hydrogen bonds.^105−107^ Bound water can also act as a plasticizer and allows both sliding, and more importantly, restabilization after deformation.^107^ Such properties are key to enabling plastic deformation.

Adsorbed water on cellulose materials has also been considered an obstacle to the chemical modification of cellulose surfaces. Water can hinder some chemical reactions by competing with the hydroxyl groups of cellulose for the reagents. However, it has been recently proven that confined water in the nanopores between cellulose fibers can enhance the acetylation of cellulose surfaces.^108^ Thus, the natural hygroscopicity of cellulose should not always be seen as a negative property. In this line, there are several attempts to exploit the cellulose–water interactions in advanced materials. Examples include cellulose-based humidity sensors and cellulose materials and composites with stimuli-responsive, shape-memory, self-healing, and adhesive properties.^38,109^ Cellulose–water interactions are also very important in hydrogels used, for instance, in biomedical applications. These are discussed more thoroughly in section 3.4.

Recently Leppänen et al.^72^ demonstrated the advantage of the hygroscopicity of nanocellulose networks for the entrapment of nanoscaled plastic particles from aqueous dispersions. Interestingly the binding of the plastic nanoparticles was not dependent on any specific chemical interaction. Instead, they showed, with a combination of surface-sensitive methods, nanomicroscopy, and modeling, that the governing factors were the high active surface area and high hygroscopicity of the nanocellulose films, the latter inducing strong capillary flow.

Clearly, the interaction of CNMs with water and ionic solutes is relevant in many practical applications and, consequently, significant efforts have been made to understand these. However, less attention has been given to interactions with other media addressed in section 3.2.

### 3.2 Interactions of CNMs with Nonpolar Solvents

In the previous section, we learned how cellulose interacts strongly with water and the implications it has on CNM performance in applications. For the same reasons, that is, the high abundance of hydroxyl groups at the surface of CNFs and CNCs, they are poorly dispersible in nonpolar media. Hence chemical functionalization of CNF or CNCs in nonpolar organic solvents like toluene has been challenging, and so reactions that can be performed in aqueous media have been the preferred choice. The reason is that, due to the poor compatibility with nonpolar solvents, the CNMs tend to aggregate and lose their nanostructure. Johansson et al. demonstrated that silylation of CNF in an amphiphilic solvent, dimethylacetamide, resulted in a surface substitution of 0.9 in comparison to only 0.03 in toluene, confirming this hypothesis. For a more comprehensive review on CNF surface modifications, the reader is referred to Missoum, Belgacem, and Bras.^110^ Here we discuss the topic mainly from the point of view of interactions between CNMs and the media.

In one of the seminal papers on cellulose nanopapers, Henriksson et al.^111^ noted that the solvent affected density and porosity of the nanopapers and consequently their strength. The densest and strongest nanopapers were formed from aqueous CNF dispersions, while less polar solvents like methanol, ethanol, and acetone resulted in more porous and slightly weaker films. This phenomenon was explained to be caused by the weakening of interfibril bonds due to reduced hydrogen bonding density when films were prepared from less polar liquids. This observation is in line with the hypothesis of Johansson et al. of the tendency of amphiphilic cellulose to adapt its conformation to the media.^75^

Tuning the interaction with the media is important in many applications, and the ability of some solvents to deswell CNF hydrogels has been applied both in nanocomposite preparation and wet spinning of cellulose filaments.^84,112^ Capadona et al.^112^ slowly exchanged water with acetone in a CNC dispersion. This led to densification of the CNC network and gelling. When a polymer solution was subsequently added, and the nanocomposite was dried, surprisingly good mechanical properties were achieved due to an even distribution of the CNC throughout the polymer matrix using this sol–gel approach. While even distribution of the components in composites is a prerequisite to achieve adequate mechanical properties, favorable interactions between fibrils and matrix polymer are needed to gain full advantage of the unique properties of CNF or CNCs. This is discussed further in section 3.3.

Unfortunately, there are very few studies on how CNMs interact with solvents. One positive exception is the work by Wang et al.,^84^ in which they investigated the influence of different coagulation agents (organic solvents and aqueous electrolytes) on the spinnability of TOCNF suspensions using QCM-D. They observed a significant increase in the resonance frequency (Δ*f)* and a decrease in dissipation factor (Δ*D*) upon introducing ethanol to the water-swollen TOCNF film. This response can be either due to the exchange of water with less dense ethanol or due to deswelling because of poorer interaction between cellulose and ethanol. Most probably, the observed response was due to both effects. More efforts should be put into exploring CNM interactions with other-than-aqueous media using surface-sensitive techniques. However, QCM-D is very sensitive to the density and viscoelastic properties of the solvents, hence care should be taken to also record the bulk effects using pure gold crystals, for example, before coating with CNMs to enable decoupling between bulk solvent effects and interactions between solvent and cellulose.

### 3.3 Interactions of CNMs with Polymers

A huge effort is being made in the development of cellulose-based materials to replace synthetic, oil-based products in a wide range of applications, including textiles, packaging, and barrier materials. In many of those cases, CNMs are combined with different polymers with the aim of obtaining composites with superior properties. In this research area, a fundamental understanding of the affinity and surface forces between polymers and cellulose is crucial for the successful design of cellulose composites with tailored properties. Deep comprehension of polymer–cellulose interactions at the molecular level is also very important for the traditional paper industry, where cationic polyelectrolytes are commonly used to flocculate cellulose fines and particle fillers. The success of current trends in replacing oil-derived additives with biopolymers in paper manufacturing and coating will benefit from a thorough understanding of the cellulose–biopolymer interactions.^113,114^

CNMs are commonly used as reinforcing components in polymer composites. However, blending hydrophobic polymers with cellulose nanomaterials is tricky because the abundant hydroxyl groups on the cellulose surfaces lead to poor polymer–cellulose compatibility. To enhance the affinity of hydrophobic polymers for cellulose nanomaterials, different strategies have been applied involving chemical modification of cellulose surfaces or covalent attachment of polymers.^115^ Nevertheless approaches avoiding hydrophobization of cellulose surfaces or covalent binding of polymers are often preferred for greener and more sustainable solutions. In this context, the utilization of natural polysaccharides or cellulose derivatives in cellulose-based composites has attracted considerable interest.

Nonionic cellulose derivatives have been observed to adsorb on cellulose substrates to a different extent, which may have interesting applications in textiles.^116,117^ Nevertheless they are not the only nonionic polymers investigated in relation to cellulose materials. Inspired by the close association between hemicellulose polysaccharides and cellulose fibers in the plant cell walls, several studies have been carried out to better understand hemicellulose–cellulose interactions for the development of natural composites.

QCM-D and SPR analyses have shown that nonionic polysaccharides of the hemicellulose family, xyloglucan (XG), galactoglucomannan (GGM), arabinoxylans, and galactomannans, adsorb well and irreversibly on CNF films (Figure 2b).^89,118−120^ The adsorption of XG and other nonionic polysaccharides on CNC has also been reported.^121,122^ The amount and conformation of the polysaccharide in the adsorbed layers do not depend only on the polysaccharide molecular weight^123−126^ but also on their concentration and molecular structure.^89,118,122^ Thus, Villares et al.^122^ observed that the amount of adsorbed XG on CNC increased with the XG concentration in solution, indicating that the lateral rearrangement of adsorbed XG molecules to a flat conformation in diluted solutions was prevented when more XG molecules competed for adsorption in more concentrated solutions. Consequently more crowded adsorbed layers with loops and tails exposed to the solution were expected in the latter case.^122^ Furthermore, Eronen et al. observed that the adsorption of galactomannans on CNF decreased when the number of galactose side groups increased, showing that the molecular structure of the polysaccharide affected the adsorption more than the molecular weight.^118^ The reason for that could be a more coiled conformation of the polysaccharides with fewer amounts of galactose side groups, which eventually resulted in a larger number of GGM molecules adsorbed per unit of area. Chemical modifications of the polysaccharides can also affect their affinity for cellulose. Thus, enzymatically oxidized guar gum galactomannan (GG) was observed to adsorb quickly on CNF films, but the adsorption rate decreased when polyethylene glycol (PEG) chains were covalently grafted to the GG molecule,^119^ probably due to some steric hindrance associated with the PEG chains. On the other hand, the addition of different, short hydrophobic tails did not hinder the adsorption of GGM on CNF,^127^ while the oxidation of GGM by TEMPO decreased significantly its affinity for cellulose.^128^ The latter could be explained by electrostatic repulsions between CNF and the carboxyl groups introduced in GGM during TEMPO oxidation, as confirmed by the fact that GGM with a high degree of oxidation did not adsorb on CNF in water but adsorbed to some extent when the electrostatic repulsions were screened in the presence of 0.1 M NaCl.

**Figure 3:**
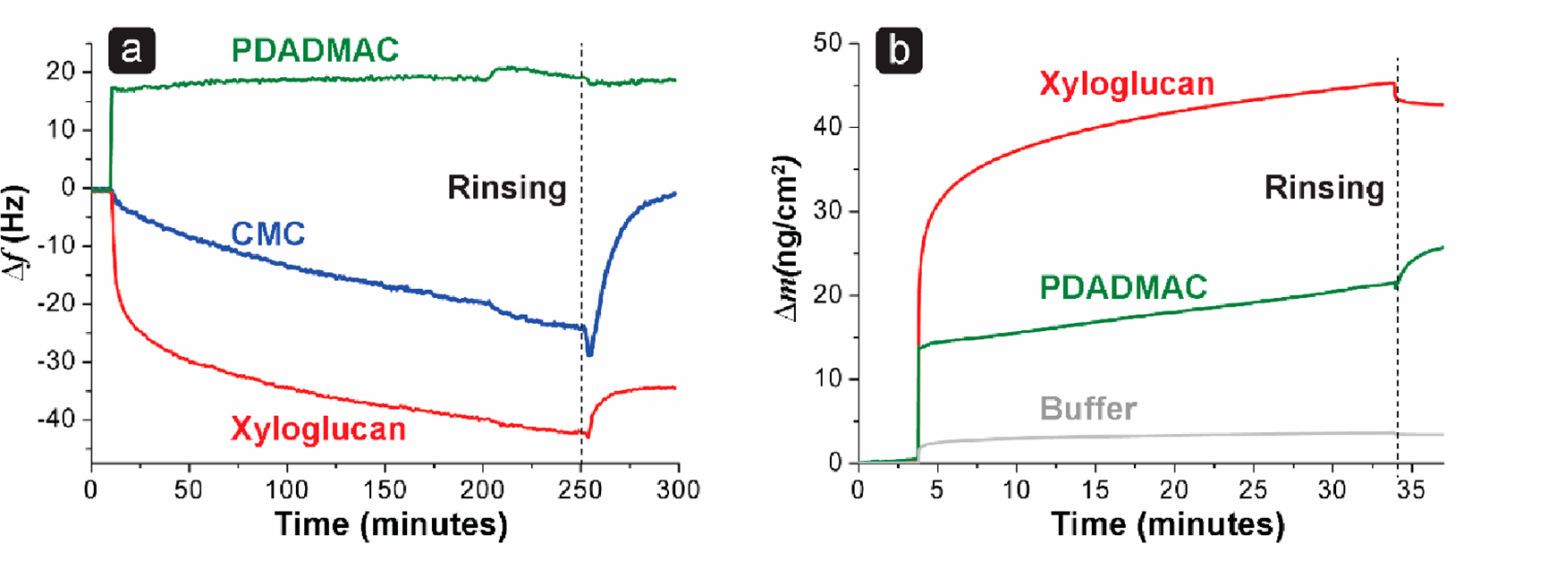
Adsorption of PDADMAC, CMC, and XG on CNF. (a) Change in frequency (Δ*f*) from QCM-D experiments, and (b) adsorbed mass (Δ*m*) from SPR experiments.^133^ Adapted from ref (133). Used under open access from BioResources.

The adsorption of nonionic polymers to cellulose has commonly been ascribed to hydrogen bonds and vdW forces. However, several authors have argued that the adsorption of nonionic polymers like XG is in fact entropically driven by the release of structured water around the polymer and the cellulose surface. Hydrogen bonds can be formed after the polymer is adsorbed, but their contribution to the adsorption process is negligible.^129−132^ The driving force for the adsorption of anionic polymers is also expected to be entropically driven due to the release of water and counterions, but in this case, the polymer charge also plays an important role. Thus, anionic polysaccharides like xylan (hemicellulose) and carboxymethyl cellulose (CMC) have been observed to adsorb on CNF to a lesser extent and form more swollen (hydrated) layers than nonionic polysaccharides (Figure 3a).^118^ In line with the assumption that the electrostatic repulsions with the negatively charged cellulose substrates can prevent or weaken the adsorption of highly charged anionic polymers, CMC was observed to adsorb irreversibly to CNF at pH 4.5,^118^ but it desorbed upon rinsing at pH 8 when all of its carboxyl groups were deprotonated.^133^ The swelling of the adsorbed polymer layer also depends on the polymer charge. Anionic polymers swell to a larger extent than nonionic ones because of the electrostatic repulsion between polymer charged groups and the osmotic pressure associated with the accumulation of counterions around the charged polymers. Therefore, in contrast to nonionic polymers, factors like the pH or the ionic strength have a very pronounced effect on the adsorption and the swelling of ionic polymers. An interesting case is the effect of divalent cations on the adsorption of CMC on cellulose substrates. Ca^2+^ ions have been observed to affect CMC structure in solution and favor the CMC adsorption on cellulose more than Mg^2+^ ions (Figure 2c,d), which was ascribed to the different polarity of those ions.^90,134^

Although also affected by the pH and the ionic strength, the adsorption of cationic polymers on cellulose surfaces is driven by attractive electrostatic interactions between oppositely charged groups and, especially, the remarkable gain in entropy associated with the release of bound counterions.^135^ The adsorption of cationic polymers like poly(diallyldimethylammonium chloride) (PDADMAC) and chitosan on CNF has been monitored in real-time by QCM-D and SPR.^118,133^ At low pH and ionic strength, chitosan has been observed to adsorb on CNF in lower amounts than nonionic polysaccharides, suggesting a flat conformation for the adsorbed chitosan molecules.^118^ Adsorption in flat conformation is generally expected in conditions where the electrostatic attraction between polymer and cellulose is enhanced, that is, at low ionic strength and pH values where the involved groups, both on the cellulose surface and in the polymer, are charged. Furthermore, a collapse of CNF films by the release of trapped water is often observed upon adsorption of cationic polymers, which is observed as an increase in frequency in QCM-D (Figure 3).^133,136^ Dehydration of cellulose surfaces and screening of electrostatic repulsion between charged cellulose fibrils could explain the collapse of CNF films induced by cationic polymers.

The preparation and the final macroscopic properties of composites and hydrogels are intimately connected to the surface forces between the constituting materials at micro- and nanoscales. Due to the large area-to-volume ratio of CNF and CNC, surface forces play a very important role in the formation and behavior of composites and hydrogels that include those CNMs. The surface forces govern the colloidal stability of CNM suspensions and, consequently, affect their rheological behavior. Thus, rheological measurements can provide indirect information on the stability or aggregated state of CNM suspensions and how factors like the CNM concentration, the ionic strength, or the presence of other polymers affect the interactions.^137,138^ Nevertheless the direct quantification of surface forces has only been possible thanks to very sensitive instruments like the surface force apparatus (SFA) and AFM.^139,140^ The SFA and the AFM, especially in combination with the colloidal probe technique,^141^ have tremendously advanced our understanding of the surface forces in lignocellulosic systems.^142^ Thus, it has been observed that the interaction forces measured when approaching different cellulose model surfaces are generally well described by the DLVO theory at long separations, whereas a steric repulsion usually appears at short distances when the cellulose surfaces come into contact.^143^ The intensity and range of the repulsive double-layer forces increase with the cellulose surface charge, and they decrease when increasing the ionic strength, as the DLVO theory predicts.^133,144^ Attractive vdW forces between cellulose surfaces have been detected in conditions where the double-layer repulsion was negligible.^144,145^

The adsorption of polymers affects the surface forces between cellulose substrates. In fact, very different interaction forces can arise depending on the amount and conformation of the adsorbed polymer. DLVO forces are typically observed when a cationic polymer adsorbs in flat conformation on cellulose surfaces, with the double layer repulsions modulated by the extent of surface charge neutralization or reversal caused by the adsorbed polymer. This behavior is typically the case of highly charged, cationic polyelectrolytes like PDADMAC, polyvinylamine (PVAm), and PVAm derivatives.^146−148^ On the other hand, nonionic polysaccharides, anionic polymers like xylan and CMC, and cationic polyelectrolytes with low charge density and high molecular weight like cationic polyacrylamide (C-PAM) usually adsorb on cellulose surfaces in an extended conformation with loops and tails. This extended conformation results in long-range steric repulsions when the polymer molecules adsorbed on two approaching surfaces overlap and compress each other.^149^ The intensity and range of the electrosteric repulsion are directly related to the swelling of the adsorbed polymer layer. The larger the swelling of the adsorbed layer, the longer the range and stronger the intensity of the electrosteric repulsion are.^119,146,150−153^ Because the pH, the ionic strength, and the polymer concentration affect the swelling of adsorbed charged polymers, the electrosteric repulsion can be modulated by changing those magnitudes.

Cationic polymers are typically used to increase paper strength and as retention aids to flocculate cellulose fines and mineral filler particles with cellulose fibers in papermaking. Several works have been devoted to the analysis of the forces between cellulose surfaces in the presence of different cationic polymers to shed light on the mechanisms underlying papermaking processes.^146−148,153−155^ In addition to the forces observed on approach discussed previously in this section, the adhesion measured when retracting the surfaces is especially relevant in this case. Both concentration and conformation of the adsorbed polymer have important impacts on the adhesion between cellulose surfaces and between cellulose and mineral surfaces like mica, silica, or glass used as models for filler particles. The partial coating of the surfaces at low polymer concentrations gives rise to adhesion by charge neutralization or polymer bridging. Polymer bridging is enhanced in the case of polymers with high molecular weight adsorbed in an extended conformation. However, increasing the polymer concentration results in strong electrosteric repulsions and no adhesion between fully coated surfaces, confirming that polymer overdosage should be avoided for effective polymer-induced flocculation.^146,153,154^ Polyelectrolyte complexes formed by the combination of cationic and anionic polymers have been observed to enhance the adhesion between cellulose surfaces, in line with empirical observations in the paper industry.^156^ The combination of colloidal assemblies of a cationic block copolymer with carboxymethylated CNF has also been explored as an alternative strategy for the preparation of biomimetic nanocomposites.^157^

**Figure 4:**
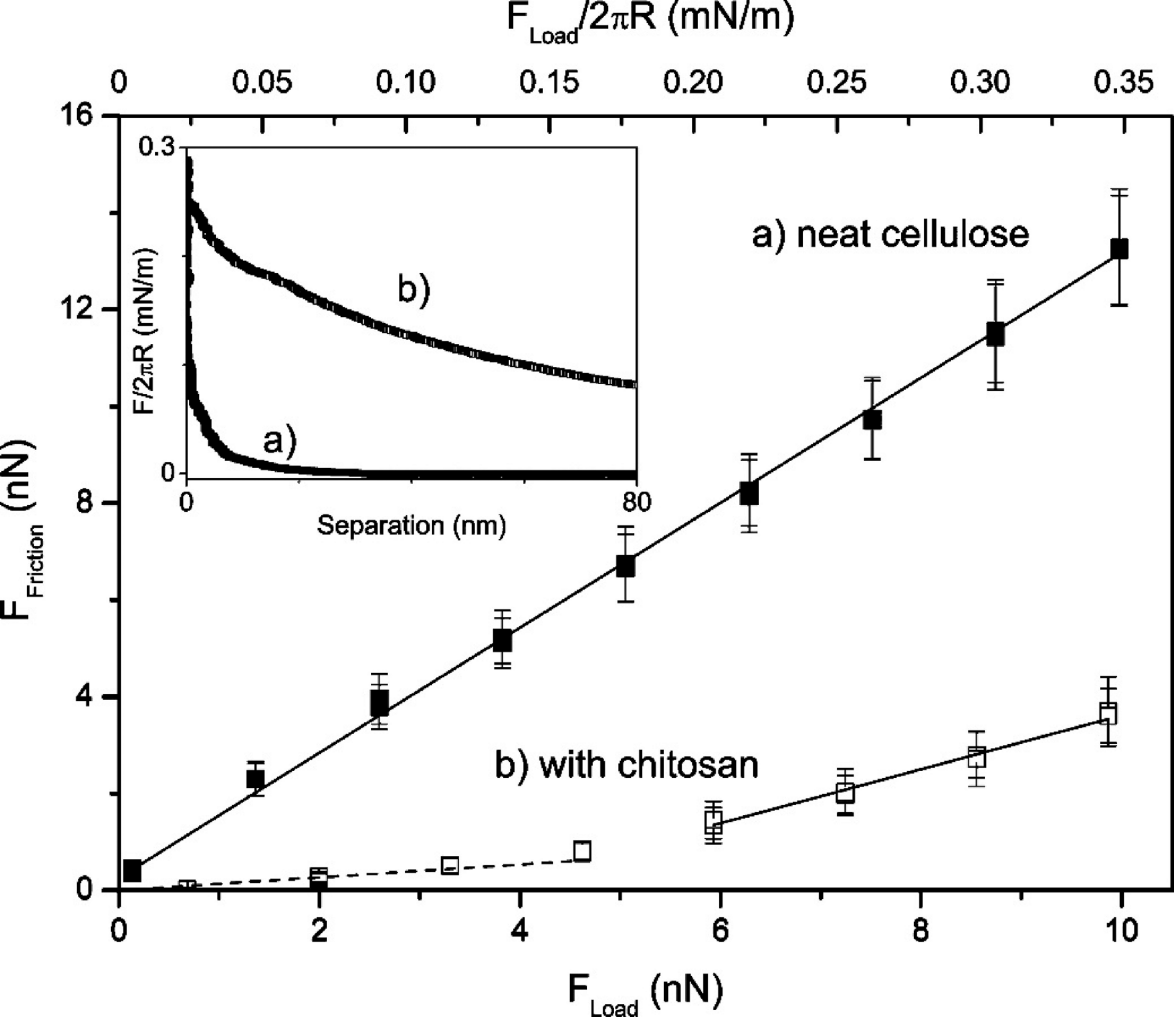
Friction measurements between (a) two neat cellulose spheres (closed squares) and (b) after adsorption of chitosan for 8 h (open squares). Measurements were conducted in aqueous solutions at pH 3. Inset: corresponding normal approach force profiles on a linear scale.^150^ Reproduced with permission from ref (150). Copyright 2009 American Chemical Society.

Friction forces at micro- and nanoscales also play a very important role in material properties. The mechanical performance of CNF-based materials, for instance, is highly dependent on the friction between cellulose fibrils. Quantitative measurements of friction forces using an AFM and the colloidal probe technique have revealed that, in general, the adsorption of polymers reduced the friction between cellulose surfaces. A correlation between surface forces, adhesion, and friction can be established. Low friction forces have been measured between surfaces with strong repulsion and weak (or lack of) adhesion. Thus, swollen, hydrated polymer layers adsorbed in an extended conformation enhance the lubrication between cellulose surfaces. Remarkably low friction coefficients have been obtained with highly charged anionic polymers like CMC, CMC with grafted PEG (CMC-PEG), and hyaluronic acid at pH and ionic strengths where the adsorbed polymer layers are charged and very swollen, which is associated with strong electrosteric repulsions and no adhesion between the surfaces.^151,158,159^ Additionally, CMC-PEG was shown to reduce the adhesion and friction between cellulose surfaces in dry conditions.^160^ The cationic polymer chitosan has also been observed to reduce considerably the friction between cellulose surfaces at pH 3 when the repulsion induced by the fully charged polymer is stronger (Figure 4).^150^ Thus, charged polymers can be utilized to prepare highly lubricating cellulose materials that could be used, for instance, as implants to replace damaged cartilage.^158,159^

Nonionic polysaccharides like XG, GGM, or modified GG can also reduce the friction between cellulose surfaces, but to a lower extent than highly charged polymers.^119,149,161^ Nevertheless, the moderate lubrication combined with certain adhesion provided by these polymers has been observed to be beneficial for the mechanical properties of CNF-based composites. The interfibrillar lubrication favors the homogeneous distribution of CNF during composite formation (preventing CNF aggregates or clusters that are detrimental to the composite strength), whereas the adhesion helps to keep the fibrils together, contributing to material integrity. Thus, the addition of only 2 wt % on nonionic (XG, GGM, or modified GG) or anionic (CMC) polymers have been seen to significantly improve the tensile strength and toughness of CNF films in dry conditions, in agreement with a more even distribution of CNF in the film aided by polymer lubrication.^119,162,163^ However, very different trends have been observed in wet conditions. The high lubrication and null adhesion between cellulose fibrils induced by CMC adsorption resulted in very poor mechanical integrity of CNF–CMC films in aqueous media.^162^ On the contrary, XG, GGM, and chemically or enzymatically modified GG improved the mechanical properties of CNF films in wet conditions. The highest tensile strength and toughness values were obtained for CNF–GGM films, indicating that the lower the water content of the adsorbed polymer layer, the better the mechanical properties in aqueous media.^119^

The formation of multilayers through the sequential adsorption of oppositely charged polymers or nanoparticles (layer-by-layer deposition) is a useful approach for controlled surface modification. This approach has been applied to modify cellulose substrates for different applications. For example, highly hydrophobic CNF films and cellulose textiles were obtained after depositing poly-l-lysine and negatively charged wax nanoparticles.^98^ On the other hand, the sequential adsorption of poly(amideamine) epichlorohydrin (PAE) and CNF on pulp fibers has been observed to improve paper strength, but the adsorption of preformed PAE-CNF aggregates did not.^164^ QCM-D has been successfully employed to monitor, in real-time, multilayer formation using cationic and anionic cellulose derivatives, chitosan, cationic starch, polyethylenimine, poly(allylamine hydrochloride), C-PAM, PDADMAC, CNC, CNF, and cationized CNF.^68,160,165−167^ The structure of the multilayers (adsorbed material and swelling) can be tuned by the number of adsorbed layers, the charge density of polyelectrolytes or nanoparticles, and the pH of the medium. Accordingly, the intensity and range of the measured electrosteric repulsions have been seen to correlate with the thickness and swelling of the multilayers.^68,165,167^ In some cases, attractive bridging forces have been detected when the swelling of the multilayer led to the exposure of underlying polymer layers.^68,168^

As can be observed from the works cited here, a considerable amount of research has been dedicated to understanding the interactions of CNMs with polymers at the molecular level and the surface forces at the nano-/microscale responsible for the macroscopic properties of CNM-based materials. That knowledge has a direct impact on the optimization of industrial products and processes. Nevertheless, there is still work to do in this field. In particular, environmental concerns urge for the utilization of more sustainable raw materials to replace the oil-derived additives commonly used in paper manufacturing and coatings for cellulose-based barrier materials. Biopolymers like cellulose derivatives, hemicellulose, starch, chitosan, and other polysaccharides are very good candidates to replace fossil additives, but their widespread utilization at the industrial scale has generally been hindered due to their poor resistance against water, lack of optimized industrial processing technology, and relatively higher costs with respect to fossil-based additives.^113,114,169−171^ Chemical modification of the biopolymers and a deeper understanding of their interactions with cellulose and CNMs could boost the industrial utilization of biopolymers from natural resources. The research already carried out on the interactions of hemicellulose and other natural polysaccharides with CNMs provides very valuable information to advance that path.

### 3.4 Interactions of CNMs with Proteins and Cells

Lignocellulose is naturally degraded by fungi, bacteria, or protozoans through the action of different enzymes. This degradation can be utilized in industrial processes, for example, for fuel production, and hence it is of interest to investigate the adsorption of enzymes onto cellulose. Cellulases (including endoglucanases, cellobiohydrolases, and β-glucosidases) and lytic polysaccharide monooxygenases (LPMO) can decompose cellulose following different routes.^172,173^ The synergistic combination of cellulases and LPMO can efficiently degrade cellulose fibers into glucose molecules. The presence of carbohydrate binding modules in some of these cellulose-degrading enzymes enhance their selective attachment to cellulose substrates.^174^ The adsorption and degradation activity of several enzymes on different model cellulose films have been monitored in real time using different techniques, including QCM-D, SPR, ellipsometry, AFM, and fluorescence-confocal microscopy.^175−183^

Under controlled conditions, cellulases and LPMO can be used to produce CNMs from cellulosic biomass.^184−188^ The enzyme-aided production of CNMs is more environmentally friendly than other common procedures because it does not require harsh chemical reactions (e.g., acid hydrolysis or chemical oxidation) nor intensive mechanical fibrillation. Cellulose degrading enzymes could also be used in applications where CNM constructs are meant to be disintegrated at the end of their lifetime, for example hydrogels or 3D scaffolds for some biomedical applications.

The utilization of CNMs in biomedical applications has been intensively explored in the last two decades. The natural hydration of cellulose hydrogels, their mechanical properties, and their ability to adsorb or encapsulate different molecules are attractive characteristics for drug delivery and wound healing or 3D scaffolds for tissue engineering. Although some studies have reported a certain level of pulmonary inflammation and toxicity upon exposure to CNMs (especially in the case of CNC), numerous works have confirmed that CNMs, and CNF in particular, are nontoxic and biocompatible materials.^189−191^ The animal-free origin of CNMs has also been an advantageous property for biomedical applications. Plant-derived CNF or BNC has been successfully used in 3D cell cultures and 3D printed bioink scaffolds either alone or in combination with other polymers and nanoparticles.^192−199^

**Figure 5:**
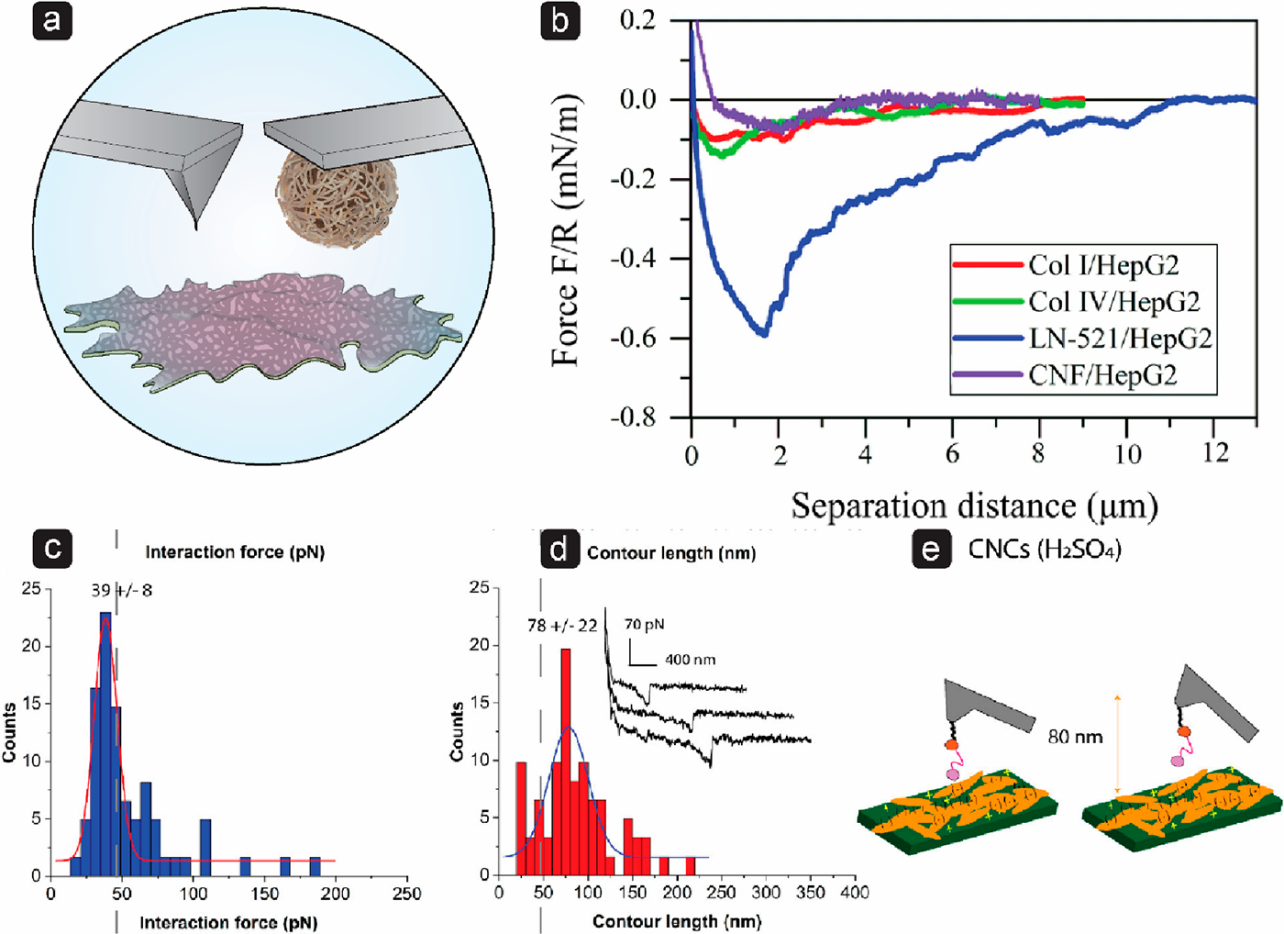
(a) Schematic of interactions between biomaterial-coated probes and living cells: a typical probe with a tip (left) and a colloidal probe (right). (b) Representative retraction curves on HepG2 cells.^200^ Reproduced from ref (200), under Creative Commons CC-BY license. Single-molecule force spectroscopy (SMFS) histograms representing (c) the binding interaction force and (d) contour length between cellulose binding protein module CBM1 and CNCs from sulfuric acid hydrolysis. (e) Schematic of the SMFS experiment for the system studied.^202^ Reproduced from ref (202), under Creative Commons CC-BY license. Copyright 2019 American Chemical Society.

The structure and surface chemistry of a material affects its interaction with cells. Although CNF hydrogels can mimic the fibrillar structure of the extracellular matrix (ECM), the polysaccharide nature of cellulose is very different from the protein nature of ECM. Consequently, AFM measurements applying the colloidal probe technique (Figure 5a) have revealed that the adhesion of human hepatocellular carcinoma cells (HepG2) and human pluripotent stem cells to CNF is considerably weaker than to ECM proteins like collagen I, collagen IV, and laminin-521 (Figure 5b).^200^ The adhesion of cells to CNF was also observed to be nonspecific, that is, it is not mediated by cell receptors like integrins.^201^ Due to the low affinity of cells for CNF, this material is not a good substrate for traditional 2D cell culture.^200^ However, CNF hydrogels have proven to be good material for 3D cell spheroid formation, where the adhesion between cells is expected to be stronger than between cells and material.^193,194^

The weak and nonspecific interaction of CNFs with stem cells results in a lack of signaling for cell differentiation. Thus, CNF and BNC hydrogels have been observed to be excellent materials to keep the stemness of stem cells for several weeks.^193,203^ Nevertheless, CNF and BNC hydrogels have to be modified if stronger cell adhesion and cell differentiation are desired. Proteins from the ECM can enhance cell adsorption and differentiation through their specific interactions with cell membrane receptors, and therefore a logical strategy to extend the applications of CNF in tissue engineering is to adsorb ECM proteins on the surface of the CNFs. Quantitative adhesion experiments between CNF and ECM proteins by the AFM-colloidal probe technique have shown affinity of collagen I, collagen IV, and laminin-521 for CNF to a different extent (stronger in the case of collagen I).^204^ AFM, fluorescence microscopy, and SPR studies have confirmed that the adsorption or immobilization of collagen I, collagen IV, laminin-521, and fibronectin on nanocellulose substrates enhanced the adhesion of stem cells, fibroblasts, and HepG2 cells.^205,206^ Strong immobilization of fibronectin, vitronectin, or collagen I on nanocellulose hydrogels via avidin–biotin or covalent conjugation has also been proved to promote integrin-mediated cell adhesion and facilitate the proliferation of fibroblasts, endothelial cells, and mesenchymal stem cells.^207,208^ Incorporation of growth factors into 3D CNF scaffolds is another approach to enhance fibroblast proliferation.^209^ The quantification of the interaction forces between cellulose binding proteins (CBM1) and cellulosic nanomaterials has also been demonstrated using single molecule force spectroscopy experiments employing a combination of click chemistry and protein engineering (Figure 5c,d,e).^202^ All of these studies undoubtedly support the promising future foreseen for CNMs in tissue engineering applications.

The interaction of CNMs with bacteria and viruses has also been under research for the development of new antimicrobial materials. Different surface modification and functionalization strategies of CNMs have been proposed to achieve materials with excellent antimicrobial properties and membrane filters against microbes.^210−212^

Importantly, nanocellulose–protein interactions can be exploited beyond biomedical applications. Thus, the combination of CNC with bovine serum albumin or CNF with soy protein has been observed to stabilize emulsions (Pickering emulsions), which is of interest to the food and pharmaceutical industries, for example.^213,214^ Furthermore, proteins like casein and zein have been used to improve the mechanical properties and thermal stability of composites containing CNFs.^215,216^ Casein, soy protein, zein, gluten, and whey proteins have also been proposed to replace fossil-based additives in paper manufacturing and coatings for cellulose-based packaging materials.^113,169^ These are just a few examples of the unlimited potential of protein-modified CNMs.

## 4 Lignin Nanoparticles

Efficient valorization of a large amount of technical lignins available as side streams from the pulping industry has challenged the industrial and academic community for centuries. The transformation of technical lignins into nanoparticles offers an interesting alternative to fractionation and depolymerization. Lignin nanoparticles solve the main drawbacks of technical lignins. Their morphology can be made homogeneous, they can be used without solvents, and they have a very large surface area, which increases their capacity to interact with their surroundings.^217^ Concurrently it would be significant to find a nanoparticle formation method that offers the desired lignin properties for specific applications but is also simple and reproducible in its approach, and economically and environmentally viable. Both research and industrial communities have collaborated broadly in their efforts to produce LNMs for a variety of applications. As a result, various methods have been introduced to produce nanoscale lignin (LNPs) or colloidal lignin particles (CLPs).

The preparation of LNPs with well-defined surface chemistry, controlled nanoarchitecture, and long-term stability is important for high-value applications. LNPs fabricated using different approaches tend to present different surface morphology, size, polydispersity, surface charge, etc., which is a hallmark of its specific fabrication processes. Acid neutralization and solvent shifting have been the most frequent routes adopted to prepare LNPs, acidification being the first reported^218^ method. Several recent review articles reveal the existence of many other approaches, such as acid-catalyzed precipitation, flash precipitation, water-in-oil microemulsion methods, homogenization, ultrasonication, and sono-solvent shifting.^6,219−223^ Among the developed LNP fabrication methods, the critical analysis points to the solvent shifting approach as the method of choice due to its simplicity, viability, high yield, and excellent control over morphological features in terms of spherical geometry, with uniform size and smooth surfaces. Hence we focus mostly on that method in the following section.

Various analogous terminologies for solvent shifting like nanoprecipitation, dialysis, solvent exchange, and antisolvent process are frequently used to describe the same approach.^6^ During LNP preparations, the assembly conditions have been found to significantly affect the surface properties, mainly particle size, particle shape (geometry), surface charge, and stability.^6^ Solvent shifting and acid neutralization (also called pH shifting) have been investigated vigorously, compared to other reported methods, to elucidate the effect of different parameters on the nanoparticle’s specific surface properties.^224,225^ Multiple factors can influence the formation of the LNPs using different approaches but more precisely during solvent shifting. These include lignin source and its chemical structure, interaction with solvents, molecular weight, initial lignin concentration, dropping speed of the lignin solution, antisolvent feed rate, stirring speed, temperature, pH, and salt concentration, among others.^6^ A systematic elucidation of each parameter on LNP formation is necessary to determine the ideal synthesis conditions because these factors directly or indirectly affect the self-assembly of lignin molecules by influencing their solubility, surface charge, nucleation, and growth. This section discusses the physiochemical aspects of the formation of lignin particles, their interactions in aqueous media, customization strategies, and applications.

### 4.1 Structural Factors Affecting LNP Properties

Because LNPs are formed by the assembly of lignin molecules into nanostructures, interactions during the particle formation process strongly affect their properties. Hence factors affecting these interactions, and consequently also the final properties of the particles, are reviewed. The intrinsic chemical structure of lignin from different sources, such as hardwood, softwood, or grass, possesses different ratios of monomeric units, ultimately affecting the self-assembly process of lignin into LNPs through noncovalent forces like hydrogen bonding, hydrophobic interactions, and π–π interactions. For instance, guaiacyl units are more abundant in softwood lignin, whereas hardwood and grass lignins are rich in syringyl and *p*-hydroxyphenyl units.

**Figure 6:**
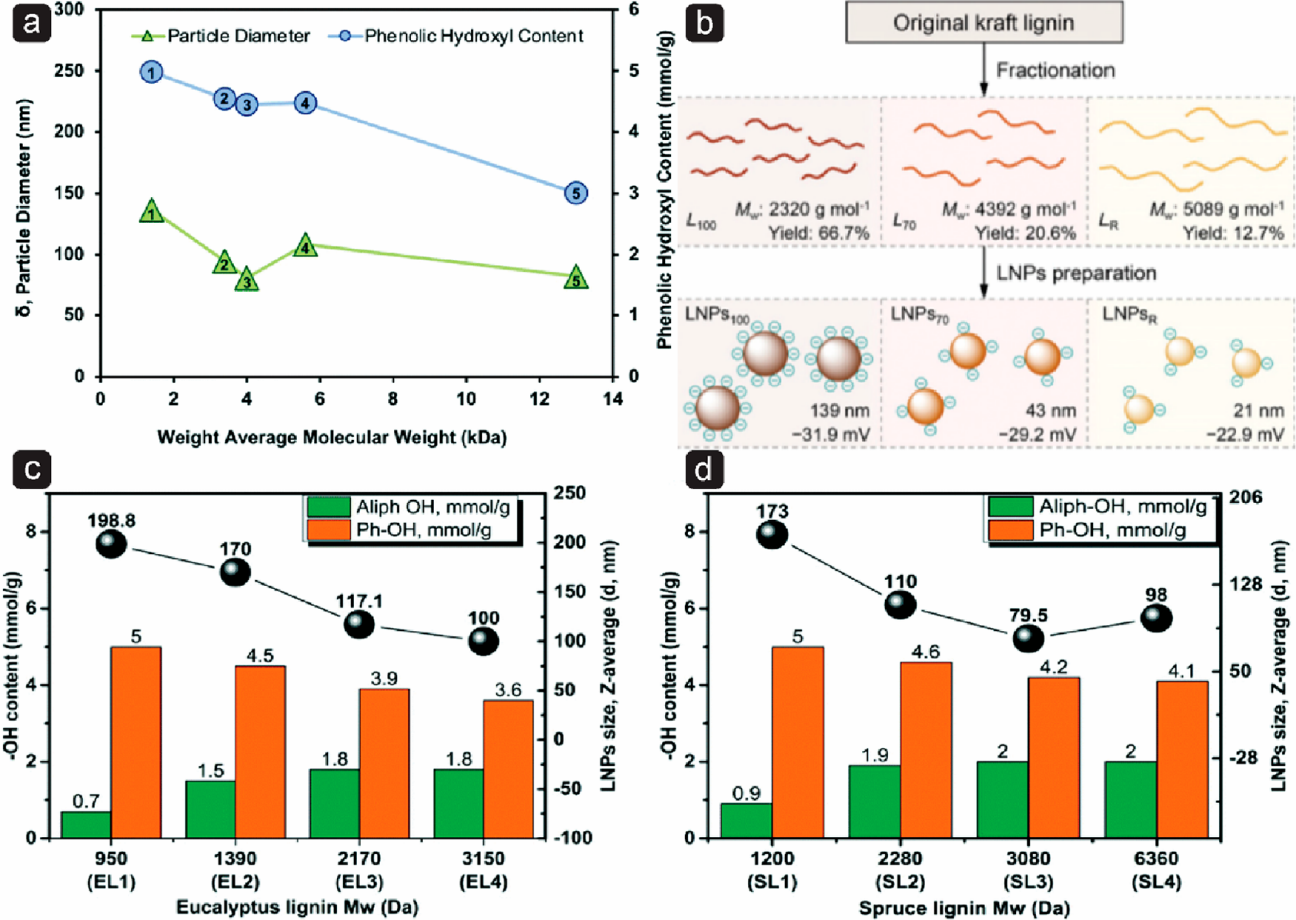
(a) Average particle diameter, weight-average *M*_w_, and Ph-OH content of different lignin fractions.^229^ Reproduced with permission from ref (229). Copyright 2021 Royal Society of Chemistry. (b) Effect of *M*_w_ on LNPs diameter and surface charge.^227^ Reproduced with permission from ref (227). Copyright Royal Society of Chemistry. LNPs diameter as a function of *M*_w_ and Ph-OH and Aliph-OH content in (c) eucalyptus lignin and (d) spruce lignin.^226^ Reproduced with permission from ref (226), used under Creative Commons CC-BY license. Copyright 2021 Royal Society of Chemistry.

Interconnected factors, such as lignin molecular weight, its solubility, and the presence of aliphatic and phenolic hydroxyl and carboxyl groups (amphiphilic nature), simultaneously affect the architecture of LNPs. Indeed the experimental evidence presented by Lievonen et al.,^217^ Figueiredo et al.,^225^ Pylypchuk et al.,^226^ Ma et al.,^227^ Pang et al.,^228^ and Zwilling et al.,^229^ all of whom studied how different lignins and different lignin fractions affect the size and morphology of LNPs, demonstrates that the particle size is decreased by high numbers of phenolic hydroxyl groups and high molecular weight. The number of phenolic hydroxyl groups depends to some degree on the lignin’s molecular weight. Low molecular weight lignin, due to the cleavage of interunit linkages (e.g., β-O-4 bonds) exhibits a high number of hydrophilic groups (e.g., hydroxyl and carboxyl groups). Similar to phenolic hydroxyl groups, lignin structures rich in carboxyl groups display higher hydrophilicity, which leads to larger sized LNPs.^230^ In contrast, the increase in the hydrophobic interactions by modifying the amphiphilic interface with *n*-alkane results in smaller LNPs.^231^ The presence of aliphatic and phenolic hydroxyl groups also plays a decisive role in LNP morphology (Figure 6). The higher number of aliphatic hydroxyl groups in the softwood lignin can result in the formation of smaller LNPs. It is postulated that the higher aliphatic content limits the lignin solubility, resulting in smaller LNPs. On the contrary, the phenolic hydroxyl groups, due to their hydrophilic nature, facilitate the hydrogen bonding between lignin molecules and water corresponding to larger LNPs size. The noncovalent π–π interactions between G-units are stronger than between S-units, so denser packing of lignin molecules during LNP formation is expected for softwood lignin, resulting in a smaller average size of LNPs.

**Figure 7:**
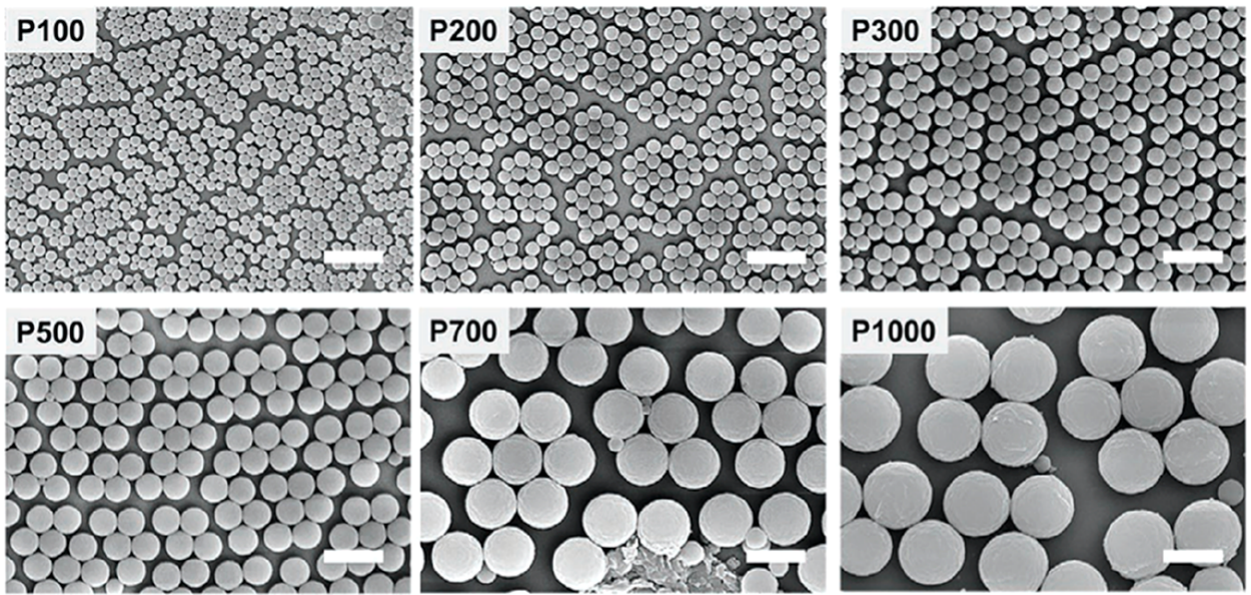
Fabrication of monodispersed LNPs with tailorable size. SEM images of a series of monodispersed LNPs (average diameters are 100, 200, 300, 500, 700, and 1000 nm). Scale bars: 1 μm.^232^ Used under Creative Commons CC-BY license from ref (232). Copyright 2022 Wiley.

The LNPs prepared in most of the reported work have rather high polydispersity. Interestingly Wang et al.^232^ were recently able to successfully prepare monodisperse LNPs with tailorable sizes (Figure 7). Their approach was based on the fractionation of enzymatic hydrolysis lignin (EHL) from corn stalk before particle formation. The EHL utilized in this work exhibited a molecular weight of 1975 g mol^–1^ and total hydroxyl and carboxyl content of 5.42 mmol g^–1^. The narrow size distribution of LNPs after solvent extraction was regarded to be a result of homogeneous intermolecular interactions between lignin molecules during the self-assembly. From the AFM force measurements, the authors concluded that the LNP sizes are dominated by long-range forces, such as vdW, electrostatic, and hydrophobic forces, rather than the short-range adhesion force like hydrogen bonding.^232^

They further observed that the lignin fraction with more carboxylic groups possesses stronger electrostatic repulsion and weaker hydrophobic force as the water content increases. In light of these observations, the authors conclude that with water content gradually increasing in the mixed solvent, the solvent properties gradually deteriorate. As a result, the attractive forces between lignin molecules dominates and LNPs are formed. They conclude that long-range interaction, especially EDL and hydrophobic forces between each lignin molecule, must be as similar as possible to obtain monodispersed LNPs.^232^

Aside from lignin chemical compositions, the correlation between the LNP size and initial lignin concentration is evident in the solvent shifting process. Typically, initial lignin concentration and LNP size exhibit a direct relationship, as the initial lignin concentration decreases, the particle diameter also decreases. This could be interpreted as due to the fact that at a low initial concentration, fewer lignin molecules are available per unit volume participating in nucleation and growth.^217,225,226,229,233−235^ Several studies using different lignin types, such as EHL,^236^ pine softwood KL,^229^ eucalyptus hardwood and Norway spruce softwood KL,^226^ and acetosolv lignin,^233^ observed an increase in size with increasing lignin concentration, which is regarded as a result of particle’s growth via the adsorption of lignin molecules to the initially formed nuclei. Besides the initial lignin concentration, the addition rate of antisolvent and the stirring speed also confer significant morphological changes on LNPs. Li et al.^233^ (acetosolv lignin powder from bamboo shoot shell), Sipponen et al.^234^ (wheat straw soda lignin), Xiong et al.^236^ (EHL), and Li et al.^237^ (KL) conclude that an increase in the antisolvent feeding rate decreased the hydrodynamic radius of LNPs and formed more nanospheres as it limited the aggregation growth time. At the same time, by increasing the stirring rate as the antisolvent is introduced, smaller LNP sizes are formed.^233,236^ It is speculated that the high stirring rate improves the mixing of organic–inorganic phases.^238^

LNP self-assembly during the acidification process is also contingent on the initial lignin concentration. A direct proportionality has been observed between the size of LNPs and their initial lignin concentration, however, unlike the solvent-exchange process, the size of the LNPs returns to a smaller size as the critical initial lignin concentration is exceeded. Frangville et al. and Agustin et al. have reported similar trends,^218,239^ although in the latter work, ultrasonication was coupled with acidification to assist the self-assembly. In both of these experimental works as well as in Gupta et al.,^240^ the increase of acidification rate (by increasing the acid’s concentration) also affected the LNP size. For instant, Frangville et al., observed an increase in particle size from less than a hundred nanometers at 0.025 M to nearly 2 mm for 2.6 M HCl.^218^ In addition, Frangville et al. and Gupta et al. reported that LNP size is reliant on the addition rate of acid into the lignin solution.^218,240^ Frangville et al. observed a 3-fold decline in LNP size, from 320 to 120 nm, upon addition of aqueous HCl at a rate of 2 drops per minute, compared to direct mixing of both phases.

The self-assembly process results in LNP surface chemistry enriched with phenolic hydroxyl and carboxylic groups. Upon dispersing in water, the charged functional groups are deprotonated, resulting in the formation of an EDL due to the counterions, which contribute to the stabilization of particles in colloidal suspension.

The reported zeta potential values of LNPs prepared from both acidification and solvent shifting methods are between −30 and −60 mV.^6,217,225,241−244^ At pH 4, the zeta potential of LNPs displays principal sigmoidal inclination, whereas a secondary inflection is observed above pH 11, which corresponds to the desorption of the more hydrophilic chains from the particles, causing a decline in surface charge.^224^ In general, the smaller the size of LNPs, the higher its zeta potential because charge density on a smaller particle is higher. Interestingly, coupling ultrasonication with acidification by Agustin et al. enhanced the surface charge of lignin, possibly by exposing the surface carboxyl or phenolic groups.^239^

### 4.2 Effect of Solvent Interactions on LNP Formation

**Figure 8:**
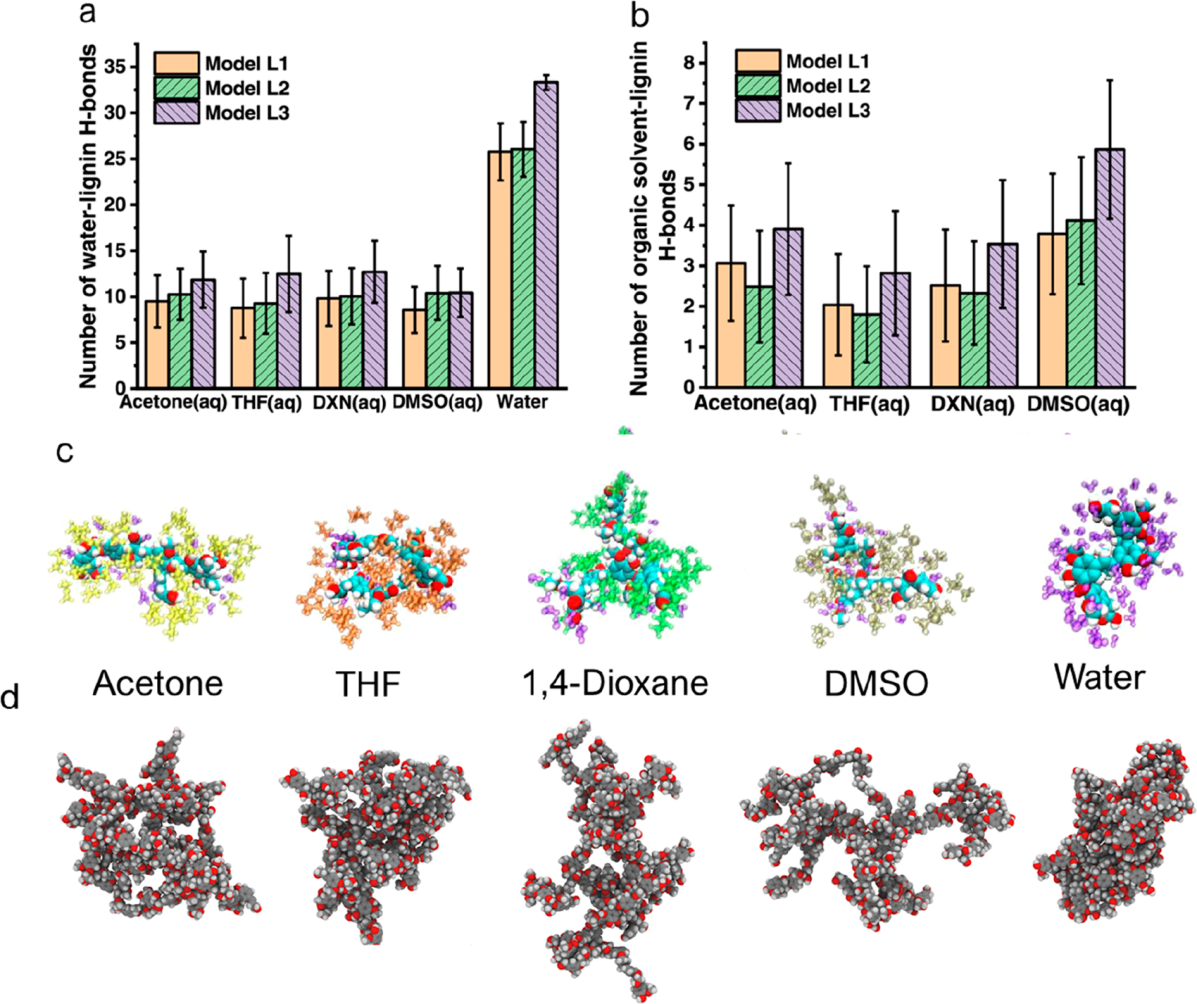
Lignin interaction with solvents. (a) Number of water–lignin hydrogen bonds and (b) solvent–lignin hydrogen bonds in different solvent systems.^247^ (c) Arrangements of solvent molecules around a lignin molecule in water–solvent mixtures with acetone, tetrahydrofuran (THF), and dimethyl sulfoxide(DMSO) as cosolvent (solvent:water = 3:1 w/w).^247^ (d) The structure of lignin in acetone, THF, 1,4-dioxane, DMSO, and water.^251^ (a–c) Reproduced from ref (247), under Creative Commons CC-BY license. Copyright 2021 American Chemical Society. (d) Reproduced with permission from ref (251). Copyright 2020 American Chemical Society.

In solvent switching methods, lignin is first dissolved in a small amount of water and a suitable low-polar organic solvent, such as acetone, tetrahydrofuran, or dimethyl sulfoxide.^217,245−247^ Because of lignin’s amphiphilic nature, binary or tertiary solvent systems containing both polar and nonpolar solvents are needed to properly dissolve lignin (Figure 8). A large amount of polar antisolvent, usually water, is then introduced. The lignin solution is usually poured into the antisolvent, but it can also be done the other way around, which provides control over the antisolvent addition rate.^217,233,234,245,248,249^ Solvent switching may also be performed by the removal of the nonpolar solvent by distillation.^250^ The change from a mostly nonpolar to a mostly polar solvent system starts a gradual aggregation process from the most hydrophobic fraction, which usually contains the largest lignin molecules,^226^ to the most hydrophilic lignin fraction.^217,234,246^ The most hydrophilic structures, being predominantly different types of hydroxyl groups, will consequently be arranged on the surface of the particles, creating a strongly negative surface charge that induces interparticle repulsion and thus colloidal stability.^217,234,247^

Because solvent interactions with lignin are important when preparing particles, the solvents affect the particle size and their inner structure. It has been found that acetone and dimethyl sulfoxide interact stronger with lignin compared to tetrahydrofuran,^247^ and particles made with acetone or dimethyl sulfoxide are smaller compared to particles made with tetrahydrofuran.^247^ The causes of these differences are still not fully known but are believed to be due to the strength of the solvent–lignin interactions.^247^ In that study, kraft lignin from soft wood was used and the experimental data was compared to molecular modeling using lignin model structures describing both kraft lignin and milled wood lignin. It is nevertheless difficult to make correct comparisons of different solvent systems. Zou et al.^247^ compared acetone, tetrahydrofuran (THF), dimethyl sulfoxide (DMSO), and 1,4-dioxane (DXN) as solvents for the preparation of lignin particles (Figure 8). Water was used as the other cosolvent. The smallest particles were obtained with DMSO, but the ratio between water and the organic solvent was not changed according to the character of the organic solvent. The optimal ratio is likely not the same for all solvents. For example, the sulfur–oxygen double bond in DMSO is more polar than the carbon–oxygen double bond in acetone or the ether bond in tetrahydrofuran, so the overall polarity of solvent systems of DMSO–water, acetone–water, and THF–water will be different when the same solvent–water ratios are used.

Solvent interactions with different structural moieties in lignin have been mentioned in publications about LNPs but rarely discussed in depth. Molecular modeling simulation studies on lignin suggest that water molecules in solvent mixtures arrange in proximity with hydroxyl groups, while organic solvents are in closer proximity with the nonpolar backbone,^247,251^ implying that the solvent mixtures are deconstructed around the solvated molecules. Solvent demixing, that is, the demixing of solvent mixtures driven by the solute’s structural characteristics, has been shown to occur in lignocellulosic biomass.^252^ Solvent demixing in the case of lignin is driven by lignin–solvent interactions, and a precise understanding of this phenomenon could perhaps be useful to tune LNP morphology and properties. However, one often overlooked factor is the interactions between the cosolvents, that is, water and the nonpolar solvent. It is known that strong lignin–solvent interactions produce small particles, but the solvent–solvent and solvent–water interactions could have an effect as well because the diffusion of a nonpolar solvent away from lignin into the bulk water facilitates particle formation. It is known that DMSO and acetone interact more strongly with water compared to THF,^253,254^ and both solvents produce smaller particles. Also, the use of THF and ethanol for particles of kraft lignin produce smaller particles than THF alone.^245^ Water and ethanol are known to interact strongly,^255−257^ and together these three solvents (water, ethanol, THF) form an azeotrope.^258^ In both of these examples, the solvent–water interactions could have an effect, but their significance has not been studied.

**Figure 9:**
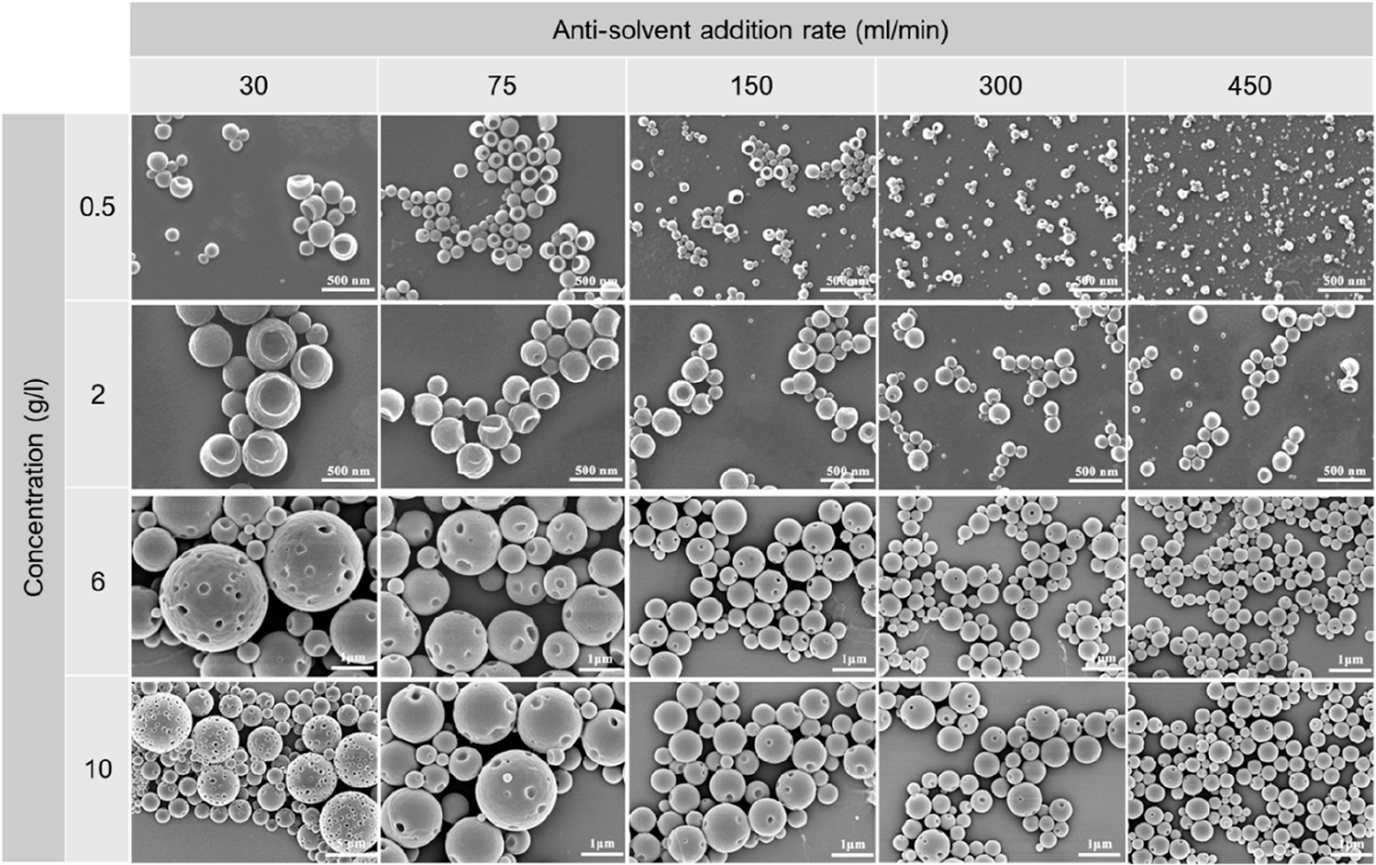
Effect of preprecipitation concentration and antisolvent addition speed on the porosity of LNPs.^233^ Reproduced with permission from ref (233). Copyright 2021 Elsevier.

The first stages of particle formation can be compared to coprecipitated hybrid particles of lignin and other amphiphilic substances.^248,259^ In coprecipitation, the more hydrophobic compound is usually concentrated on the inside of the particles where it is shielded from water.^248,259^ Likewise, it is believed that the most hydrophobic lignin structures reside within the core of the particles.^217,246^ Some studies also suggest that particles contain some amount of solvent in their core in the first stages of particle formation, thereby being a sort of nanoemulsion.^246^ The speed of the antisolvent addition has a significant effect on the particles’ structure and size, but the reason is not fully clear. Quick addition of antisolvent creates small and dense particles, while slow addition can lead to the formation of very large or hollow particles (Figure 9). Some studies suggest that the formation of hollow structures is due to hydrophobic impurities in the used solvent.^260^ However, this seems improbable to be the only cause. Hollow spheres have been obtained with various solvent systems, but only when water is added slowly to the lignin solution, and when the lignin concentration is around 2 g/L or below.^233,234,260−263^ Li et al.^233^ examined various lignin concentrations and water addition speeds using acetosolv lignin from bamboo shoot shells. They observed increased porosity and particle size with slow water addition and low lignin concentrations. It was also observed that irregularly shaped spheres are formed in low concentration and quick water addition, which is an interesting finding (Figure 9).

To illuminate some possible reasons for these findings, let us consider a few general differences in the lignin–solvent interactions throughout the particle formation in slow and quick antisolvent introduction approaches. In quick antisolvent addition, lignin molecules should readily rearrange into dense structures, where the nonpolar moieties are shielded from the antisolvent and continue to form spheres by aggregating. In slow antisolvent addition, the precipitation may be able to begin before or at the same time as the lignin molecules aggregate to shield the nonpolar structures, which could explain the reduced density. Eventually, the osmotic pressure may cause the particles to burst, which would result in pierced hollow particles. The presence of hydrophobic substances can be also used to create nanocapsules with shells made of lignin.^248,262^ Better knowledge of the reasons for differences in porosity between LNPs made by quick and slow precipitation would help to understand the aggregation mechanism, which is important to be able to precisely tailor the particle properties.

How LNPs interact with solvents after precipitation has not yet been studied extensively. QCM-D monitoring measurements have shown that LNPs dissolve by peeling in alkaline media.^264^ This is seen as a gradual decrease in particle size. However, alkaline-mediated dissolution generally requires a chemical reaction, that is, deprotonation. Organic solvents solvate polymers through physical interaction but their interaction with the LNP surface at the onset of the dissolution has not yet been studied. One existing study showed that lauric acid could diffuse out from hybrid LNPs,^248^ and multiple studies on drug release also suggest that small molecules can be diffused out of LNPs.^234,235,265,266^ This could mean that small molecules, such as organic solvents, could likewise be infused into LNPs in the right conditions. Most studies, however, focus on how to protect LNPs from dissolving in organic solvents. Both internal^259^ and external^267,268^ cross-linking have been shown to increase particle resistance against solvents. Cross-linking can be done purely chemically, but also enzymatically.^218,259,267,268^ Internally cross-linked LNPs can be used in harsh reaction conditions and allow modification of LNPs postprecipitation.^259^

### 4.3 Interactions of LNPs with Aqueous Media

**Figure 10:**
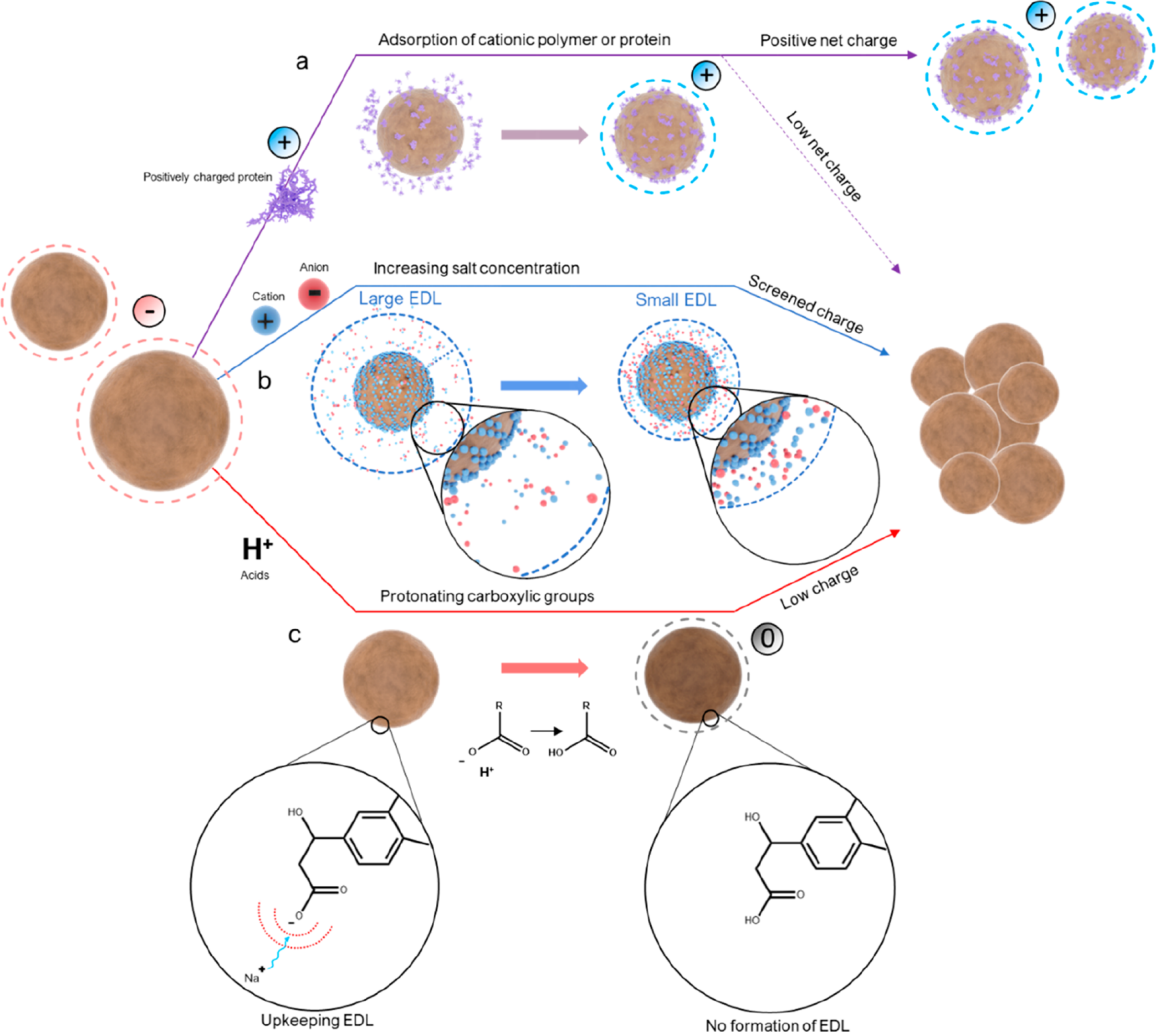
Schematic representation of interactions of negatively charged lignin nanoparticles (LNPs) with elements in aqueous media. (a) Adsorption of cationic proteins or polymers onto LNPs, leading to either charge reversal or aggregation because of reduced net charge. (b) The electrical double layer (EDL) is reduced by high concentrations of salt, leading to aggregation. (c) Protonation of carboxylic acid groups leads to charge-neutralization and thus aggregation.

The surface chemistry of LNPs determines their interaction with their surroundings. During the formation of LNPs, the most soluble structures, that is, small molecular-sized fragments containing high amounts of hydroxyl groups, are adsorbed on the particle surface at the end of the precipitation, making CLPs more hydrophilic than lignin in general.^234,264,269^ The negatively charged carboxylic hydroxyl groups on the LNP surface create an EDL, which establishes repulsion between particles.^217,234,270^ As for EDLs in general, increased ionic strength in the media reduces the range of the EDL repulsion and at high enough ionic strength attractive forces dominate and lead to aggregation (Figure 10).^217,246^ The charge of the LNPs will affect the magnitude of the EDL repulsion, and the concentration and the size of particles affects the probability of collision, hence the colloidal stability of LNP dispersions will vary depending on their properties. Clear aggregation of LNP dispersions has been observed at 1 M NaCl.^217,246^

The pH will also affect the stability of LNPs. For example, reducing pH to the isoelectric point of LNPs (ca. pH 2.0–1.5) results in aggregation because of the lost negative charge from carboxylic groups (Figure 10c).^217,271^ Likewise, LNPs dissolve when phenolic hydroxyl groups are deprotonated as the amount of strong hydrogen bonding acceptors increases, which results in stronger interactions with water.^272^ Dissolution usually starts occurring at pH levels above 10.^217,272^ Cross-linked particles can nevertheless retain their morphology also in alkaline solutions.^259^

LNP interactions with water are relevant in many applications, but there are very few studies explicitly probing these interactions.^264^ Plastic-like films from biopolymers are increasingly needed to replace plastics, and water interactions are highly relevant in this application. There are a few key problems in the development of biopolymer formulations. Perhaps most significantly, natural biobased polymers, including lignin, are too hydrophilic to provide sufficient protection from moisture. In contact with water, the inter- and intrachain hydrogen bonds are replaced with hydrogen bonds with water molecules. This logically decreases mechanical properties, especially in humid and wet conditions. In amphiphilic or hydrophobic polymer blends, the presence of water molecules between the polymer matrix and LNPs decreases the interfacial adhesion and ability to transfer load.

We encourage Nature-inspired approaches to tackle these problems, and both bound water and hierarchical structures may play important roles. As discussed in section 3.1, on its surface, nanocellulose contains bound water^95^ that can be utilized in applications like microplastics capturing or cell culture.^72^ Although the surface-bound water of LNPs has not been studied to the same extent as for CNF, the hydrophilicity and vapor sorption of LNPs indicate that they also bind water.^249,264,273,274^ Because of this, there is inevitably some water trapped within matrices containing LNPs, especially when using solvent casting to form the composite. In section 3.1, we further noted that water molecules can have a plasticizing and positive effect on mechanical properties of cellulose nanopapers. Similarly the interaction of LNPs and water should be studied more and taken into consideration in applications where LNPs are used in polar and nonpolar matrices.

In LNP–CNF films, it was observed that both cationized and regular anionic LNPs significantly increased both the stress at break and strain. This was surprising because the LNPs were expected to disrupt the hydrogen bonding pattern in CNF, however, due to the hydrophilic corona of the LNPs, they are expected to also be able to form new hydrogen bonds. The increase in strain was also surprising, because lignin is brittle, and particulate reinforcers most often *decrease* flexibility and the capacity for plastic deformation.^275^ The increased strain was speculated to be due to the LNPs acting as ball bearing and lubricating stress transferring agents. However, we cannot exclude the plasticizing effects of surface bound water. Furthermore, it was observed that solvent-casted composites were stronger compared to hot-pressed ones, which would indicate that water may have a significant effect on the strength of hydrophilic composite materials. This angle is rarely presented in the field of biomaterial composites but is discussed frequently in studies on proteins and biological systems.^107,276−278^ An interesting discussion on this topic from a biological perspective is found in Chaplin’s opinion article.^276^

### 4.4 Interactions of LNPs with Polymers

Interactions between polymeric systems can be realized at different length scales. However, when dealing with colloidal systems, the molecular level is a prerequisite from a surface chemistry perspective. The interactions of LNPs with polymers are important in different scenarios, such as adsorption of a polymer onto the LNP surfaces, dispersing LNPs into a continuous polymer phase, and in the case of Pickering emulsions, to enable better interactions with both oil and water phase. These interactions play essential roles in determining the adsorption, aggregation, adhesion, and phase separation of the colloidal particles in a resulting multicomponent system and will be discussed in more detail in this section.

**Figure 11:**
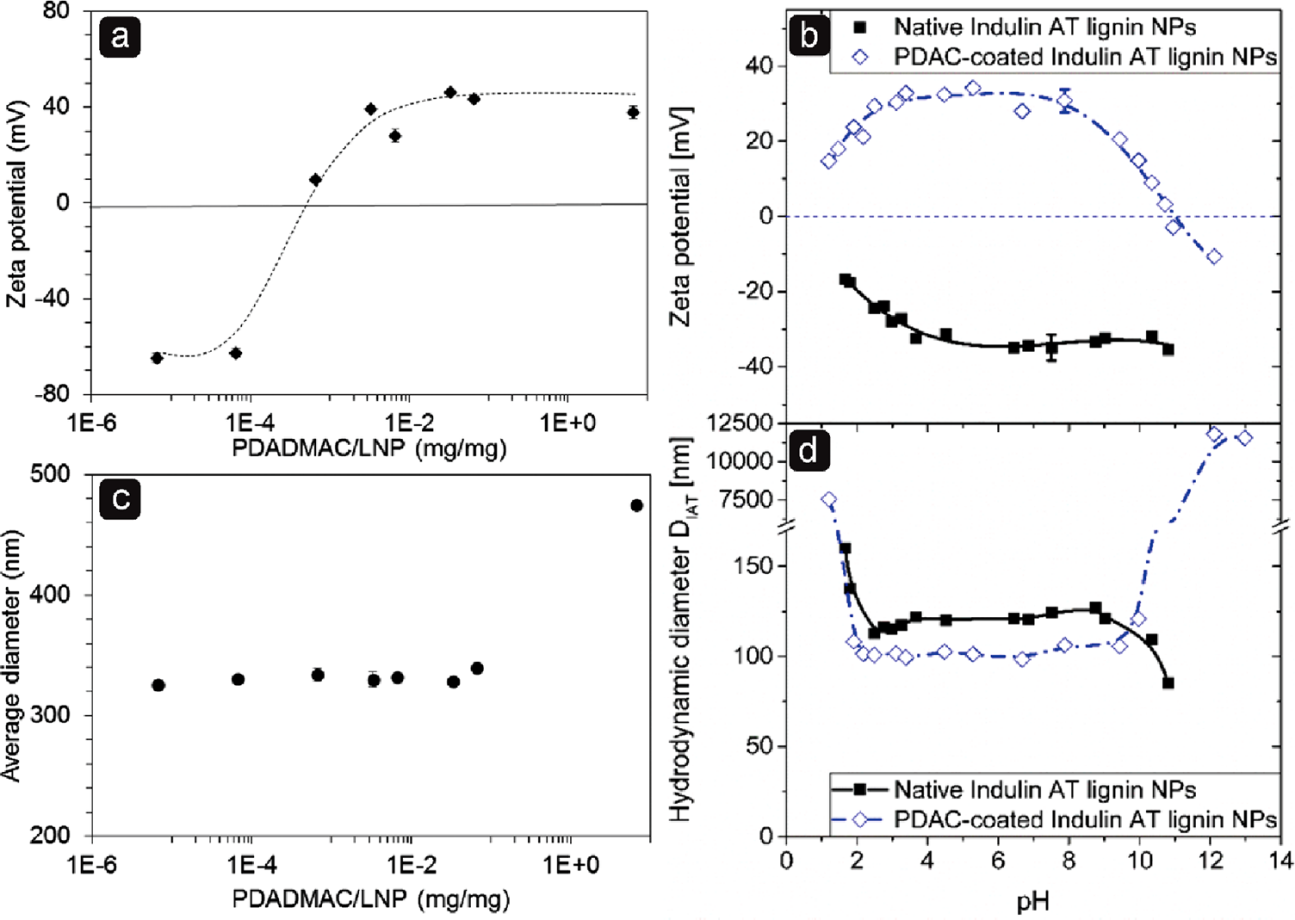
Effect of PDADMAC coating on zeta potential of LNPs prepared from KL using (a) solvent exchange,^217^ (b) acidification,^279^ and on average hydrodynamic diameter of LNPs from (c) solvent shifting and (d) acidification. (a,c) Adapted with permission from ref (217). Copyright 2016 Royal Society of Chemistry. (b,d) Reproduced with permission from ref (279). Copyright 2016 American Chemical Society.

The surface features of LNPs affecting their interactions with polymers are attributed to their negative surface charge, the presence of active functional groups like phenol hydroxyl and carboxyl, and their spherical nanoscaled dimension. The adsorption of cationic polymers onto the LNP surface predominantly relies on electrostatic interaction. To modify the affinity of LNPs to certain substrates, the adsorption of PDADMAC, chitosan, poly(allylamine hydrochloride) (PAH), and cationic lignin has been accomplished by applying the principle of electrostatic interactions, although cationic lignin is speculated to also interact with the LNPs via π–π interactions. Native LNPs exhibit a negative zeta potential, which changes from negative to positive upon the adsorption of a cationic polymer. For instance, adsorption of PDADMAC causes a charge reversal on the LNP surface as a function of PDADMAC concentration, and stable cationic particles with almost unchanged particle size are obtained (Figure 11a,b).^217^

At very high PDADMAC concentration, some aggregation occurred, most likely a result of more extended conformation of the PDADMAC chain forming tails and loops (Figure 11c). These particles were stable at pH above 4 and below 12. At pH 12, they started to dissolve. In another study using acid precipitated LNPs, a slightly different behavior was observed. At pH below 2, both native and PDADMAC modified LNPs aggregated, whereas, at alkaline pH greater than 10.5, only the PDADMAC-coated nanoparticles underwent aggregation (Figure 11d).

**Figure 12:**
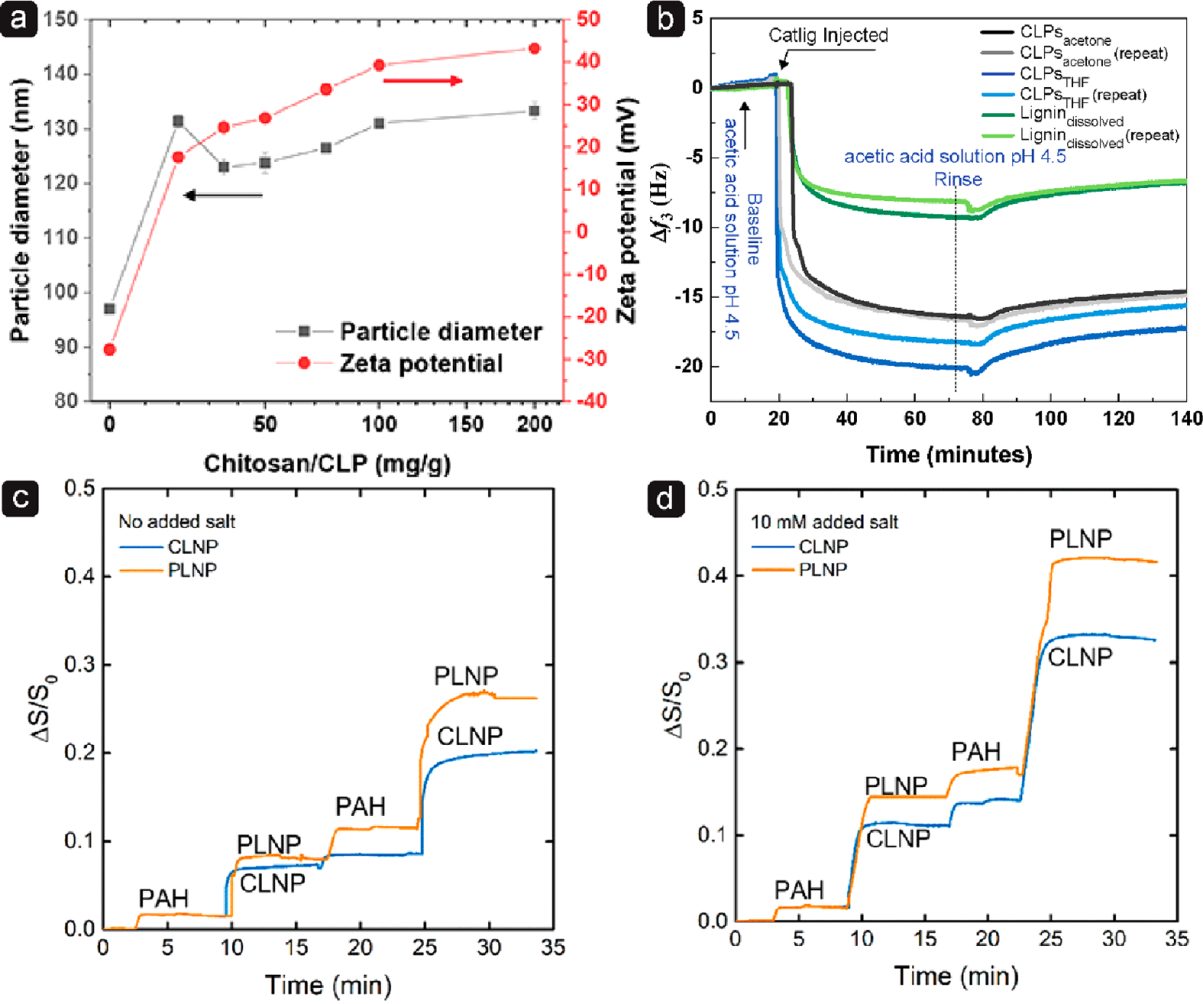
Particle diameter and zeta potential of chitosan coated LNPs (CLPs) (a) plotted against the mass ratio of chitosan to CLP.^280^ Reproduced from ref (280), under Creative Commons CC-BY license. Copyright 2019 Frontiers in Chemistry. (b) QCM-D data (third overtone) showing a change in resonant frequency upon the adsorption of Catlig onto the CLP films.^264^ Reproduced with permission from ref (264). Copyright 2020 American Chemical Society. SPAR adsorption data showing the reflectometry signal (Δ*S*/*S*_0_) for the multilayer formation of PAH (the first and third layer) and CLNP/PLNP (the second and fourth layer) onto SiO_2_ surface at (c) 0 M NaCl and (d) 10 mM NaCl.^282^ (c,d) Reproduced with permission from ref (282). Copyright 2021 American Chemical Society.

The polycationic nature of chitosan favors interactions with negatively charged microbial cell walls in addition to their emulsification capacity. Zou et al.,^280^ Moreno et al.,^296^ and Stine et al.^374^ adsorbed chitosan onto the LNP surfaces, resulting in the zeta potential of approximately +45, +32, and +34 mV, respectively, along with a slight increase in the particle diameter (Figure 12a). Although the adsorption is expected to be dominated by entropy due to the release of counterions and water molecules, hydrogen bonds between hydroxyl and carbonyl groups in chitosan and carbonyl, hydroxyl, and ether groups in lignin can be formed after adsorption decreasing the tendency for desorption.

Similar observations were made by Farooq et al.,^264^ who studied the adsorption of cationic lignin (catlig) polymer on LNPs and model lignin surfaces (Figure 12b). The adsorption resulted in a frequency shift (Δ*f*_3_) of merely 9 ± 1 Hz for the model lignin surface (Figure 12b). In contrast adsorption onto LNPs prepared from THF and acetone displayed higher frequency shifts of 19 ± 1 Hz and 16.0 ± 0.1 Hz, respectively. Authors asserted that the catlig adsorption onto the model lignin surface prepared from dissolved lignin is likely due to contributions from electrostatic, π–π, cation−π, and hydrophobic interactions, arising from the aromatic units of lignin; whereas, in the case of LNPs, the electrostatic interactions are expected to be more dominant and adsorption is driven by gain in entropy due to release of counterions and bound water. However, we need to consider that the π–π and cation−π interactions will be of importance only at <0.5 nm range. Furthermore, the nanoscale spherical geometry is also expected to enhance the Catlig adsorption to LNPs due to the larger exposed surface area. In a recent work by Alipoormazandarani et al.,^282^ the adsorption of PAH and modified LNPs was studied using a stagnation point adsorption reflectometry (SPAR) and QCM-D. They observed a different adsorption behavior for carboxymethylated (CLNPs) and carboxypentylated LNPs (PLNPs) (Figure 12c,d). For instance, PLNPs with five carbon alkyl side chains demonstrated higher adsorption capacity compared to CLNPs with one carbon alkyl side chain. It is postulated that alkyl side chain length plays a crucial role in neutralizing the opposite polyelectrolyte charges. Interestingly, the SPAR adsorption data also reveals a higher adsorbed amount of both LNPs at an electrolyte concentration of 10 mM NaCl (Figure 12d) compared to that in a salt-free system (Figure 12c). A more coiled conformation of the polyelectrolyte chain in the presence of salt is expected to enhance the adsorption due to larger charge overcompensation. The data obtained from QCM-D and SPAR complement each other, further supporting the importance of combining different surface-sensitive tools to study the adsorption of different polyelectrolytes onto a colloidal system.

In the above-mentioned examples, the adsorption of the cationic polymer was commenced directly onto the LNP surfaces to enhance their performance in applications and, also, in the case of QCM-D and SPAR experiments, to better understand the interactions of LNPs, because they have not been extensively studied yet. However, there is great interest in using LNPs in a continuous polymer phase as reinforcing, cross-linking, UV-protective, or antioxidant agents. LNPs’ large surface-to-volume ratio is expected to improve the polymer–nanoparticle interactions, resulting in more consistent biomaterials with improved properties. An array of natural and synthetic polymers has been combined with LNPs, including poly(methyl methacrylate), polyethylene, thermoplastic polyurethane phenol-formaldehyde, polybutylene adipate-*co*-terephthalate, poly(lactic acid) (PLA), poly(vinyl alcohol) (PVA), chitosan, wheat gluten, macroalgae, and cellulose. Among these polymers, PVA and PLA have been extensively studied. Wang et al.,^283^ combined LNPs with PVA/hexagonal boron nitride nanosheet and CNF, Tian et al.,^284^ utilized the combination of LNPs and PVA, whereas Yang et al.^285,286^ prepared nanocomposites and hydrogels with chitosan–PVA. In these reported works, authors associated the increase in tensile strength to the hydrogen bonding between CNF/LNPs, PVA/LNPs, and PVA/chitosan/LNPs, respectively. Yet only FTIR analysis was employed to qualitatively measure the hydrogen bonding interactions. Likewise improvements in the thermomechanical, antioxidant, and UV-shielding properties have been linked to the inclusion of LNPs in PLA-based nanocomposites. Although the LNP surface is rich with carboxyl and hydroxyl groups, which has a strong ability to form intermolecular hydrogen bonds with the carbonyl groups of PLA,^287^ the reported values for stiffness and strength were mostly in the same range as those of neat PLA, with only slight improvement.^288−291^

The loss in the ductility of the nanocomposite is also a consistent feature while integrating LNPs into polymer matrices. Nevertheless, in a more recent work, grafting LNPs with PLA and poly(ε-caprolactone) (PCL) copolymer resulted in a 6.7-fold improvement in the notched impact strength for nanocomposites, compared with neat PLA.^291^ The noticeable increase of toughness for PLA was attributed to the increased miscibility between PCL and PLLA, induced by adding a PCL–LNP–PLLA copolymer, where LNPs act as interfacial compatibilizers. It can be concluded that LNP’s surface structure is a significant factor for mechanical properties in biocomposites, hence more focus should be on choosing the most suitable particle preparation method and lignin source based on the desired properties. Hydrophobic LNPs would fit better to hydrophobic matrices and hydrophilic LNPs to hydrophilic polymers. In general, solvent switching using organic solvents create a solubility gradient that favors hydrophilic structures on the surface, while mechanical grinding,^292^ acidification,^218^ or aerosol flow drying,^293^ do not necessarily do so. Studying the strength of similar composites with different types of LNPs could thus be useful to better understand the characteristics of the particles’ surfaces in composite applications and find the best applications for different types of particles. The direct interactions between the polymers and different types of LNPs should also be assessed with adequate methods. What should also be taken into consideration in future studies is the role of water in the composites.

**Figure 13:**
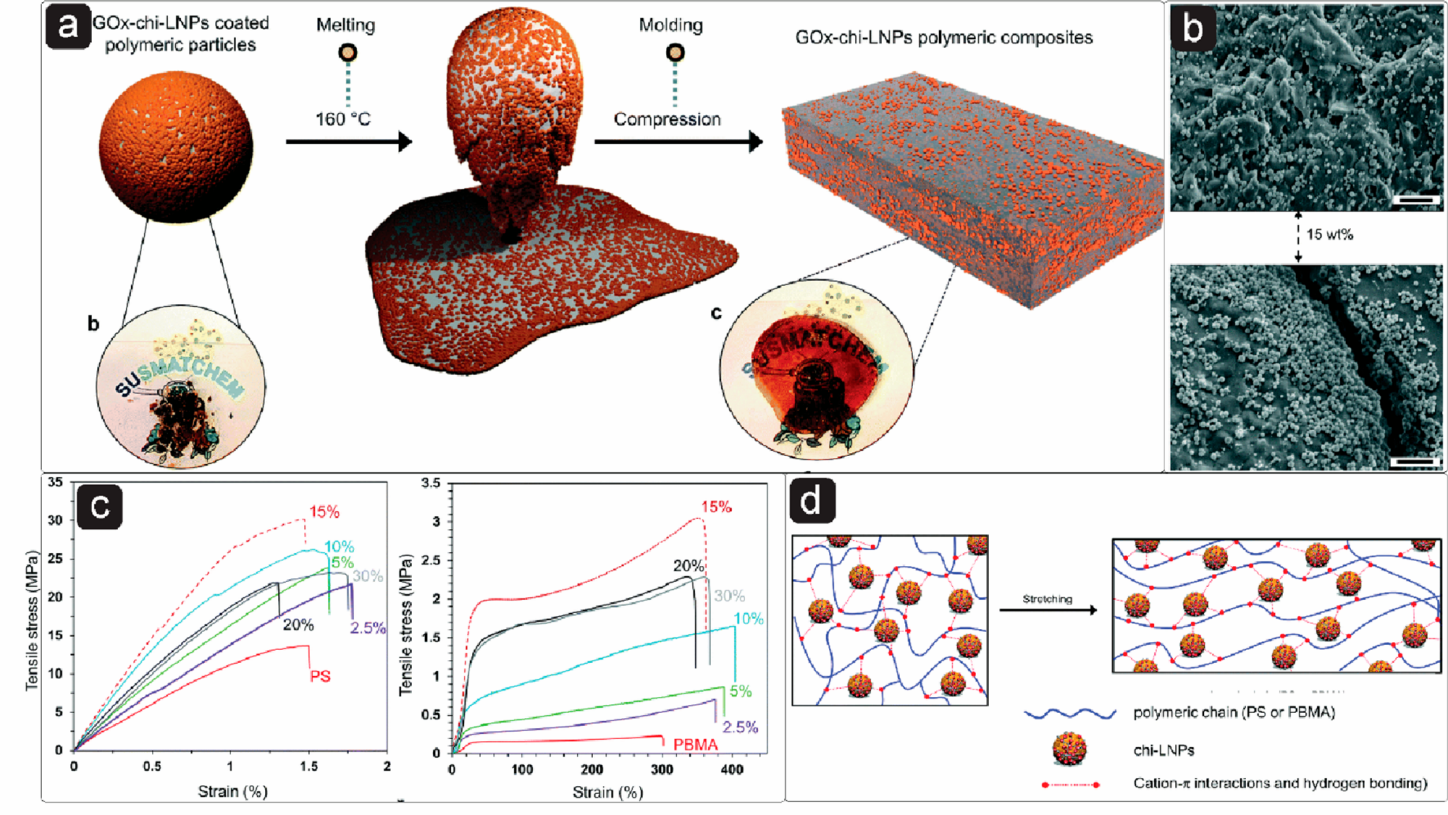
(a) Schematic illustrating the preparation of the composites by the melting process. (b) SEM micrographs of top and cross-sectional surfaces of PS-GOx-chi-LNP composite films at GOx-chi-LNPs 15 wt % . (c) Tensile stress–strain curves of pure PS and PS-GOx-chi-LNP composites and pure PBMA and PBMA-GOx-chi-LNP composites. (d) Schematic illustration of the proposed interactions between hybrid LNPs with polymeric chains before and after deformation in tensile testing.^296^ Reproduced from ref (296), under Creative Commons CC-BY license. Copyright 2021 Royal Society of Chemistry.

Due to their amphiphilic nature, LNPs are excellently suitable to stabilize Pickering emulsions in which both interactions with water and nonpolar solvents are important.^293,294^ By coating LNPs with a positively charged polymer, such as chitosan or cationized lignin, the interaction between negatively charged fatty acids can be strengthened.^280,294^ Emulsions of cationized LNPs are stable for months, which make them suitable for both cosmetics and vectors for hydrophobic substances. Pang et al.^375^ prepared composite nanoparticles from lignin/sodium dodecyl sulfate and used them to fabricate lignin/polyurea composite microcapsules through the Pickering emulsion approach. Qian et al.,^295^ prepared Pickering emulsions of decane in water with the 2-(diethylamino)ethyl methacrylate grafted LNPs. The Pickering emulsion droplets exhibited the demulsification and emulsification feature by simply applying CO_2_ and N_2_ bubbling. Sipponen et al.^294^ displayed the capability of cationic LNPs to stabilize a broad range of Pickering emulsions. Results indicated that, compared to unmodified lignin or anionic LNPs, cationic LNPs are more amphiphilic. It was anticipated that additional intramolecular, as well as intermolecular stabilization by electrostatic and cation−π interactions, can further improve Pickering emulsion stabilization. Moreno et al.,^296^ performed free radical polymerization of polystyrene and poly(butyl methacrylate using oil-in-water Pickering emulsions stabilized by hybrid LNPs coated with chitosan and glucose oxidase (Figure 13a). The hybrid LNPs dispersed homogeneously within the polymeric matrices (Figure 13b), resulting in improved tensile strength without sacrificing their elasticity in comparison to pure PS and PBMA (Figure 13c,d). The self-interactions among lignin molecules are very strong because of the large number of polar functional groups in the molecule, thus interactions play a decisive role in the determination of the structure and properties of polymer/lignin blends.

### 4.5 Interactions of LNPs with Cells

Due to the amphiphilic character of lignin, one of its obvious applications is as a carrier for hydrophobic substances, such as medicinal agents like resveratrol and benzazulene,^235,266,297,298^ anti-inflammatory agents like budesonide,^234^ or such bactericidal agents as silver particles,^279,299,300^ to reach their target. Many such substances are nonwater-soluble and therefore need some vector to be transported to their target site. With the development of new production methods, the use of LNPs in biomedicine, especially for drug delivery,^265^ has gained particular interest. There are a variety of different kinds of already commercial nanoparticles that are used in medicinal treatments, such as gold, silica, protein-based, virus-based, polysaccharide-based, dendrimers, and ceramic nanoparticles, and applications include drug-delivery (often to treat cancer), phototherapy, gene therapy, immunotherapy, and more.^301,302^ In this section, we discuss the scarce work that has to date been devoted to LNP interactions with cells as well as related work that suggest important factors to consider in future work in this area.

LNPs can be used as carrier for hydrophobic substances in a variety of ways.^265^ For example, in the same manner that LNPs can be used as Pickering emulsions to entrap oils,^280^ they can be used to make Pickering emulsions of medicinal substances.^303,304^ Medicinal compounds can also be coprecipitated with the lignin to make hybrid LNPs that contain the medicinal substance within them.^235,305^ LNPs can even be magnetized by coprecipitating the particles with iron oxide (Fe^3+^_2_O^2–^_3_), which allows for even more precise targeting under magnetic fields.^235,261,297,298^ Nanoparticle internalization has been excellently summarized by Oh and Park^306^ and therefore will not be extensively reviewed here. A general discussion of important factors is nevertheless presented here to illuminate how the properties of LNPs could be used and modified for use as drug carriers. It is also important to understand how LNPs interact with cells and to know the possible outcomes of in vivo use. This section thus addresses these interactions and the fate of LNPs when used in biomedicine.

Primarily internalization efficiency, that is, how quickly a compound is brought into a cell, is highly important to guide a medicinal agent to its target. Particles that are not internalized efficiently are, to a large extent, captured by the immune system and tend to eventually accumulate in the mononuclear phagocytic systems of central organs, such as the liver, where they can be harmful in large quantities.^306,307^ To avoid drug vectors accumulating in phagocytic cells rather than the target cells, the internalization should be optimized according to the target cell. The internalization efficiency of nanoparticles into cells is different depending on the particle size, shape, and surface chemistry and the cell type. While most nonphagocytic cell types take up nanoparticles with sizes around 50–200 nm most efficiently, phagocytic cells take up particles of 2–3 μm most efficiently but can also internalize smaller particles down to 200 nm in size.^308,309^ Non internalized particles tend to aggregate due to bridging proteins and thus grow as time progresses^310^ and may therefore eventually be internalized by the phagocytic cells.^311^ Most cell types also internalize rod-shaped particles more efficiently than spheres, which is the second most favorable shape.^306,312^ While the shape of LNPs prepared via solvent shifting cannot be altered significantly, the size can easily be tuned. Because of the size preference, small differences in size can significantly affect toxicity.^309^ Hence particles with a well-controlled and narrow size distribution should be used when applying LNPs in biomedicine.

**Figure 14:**
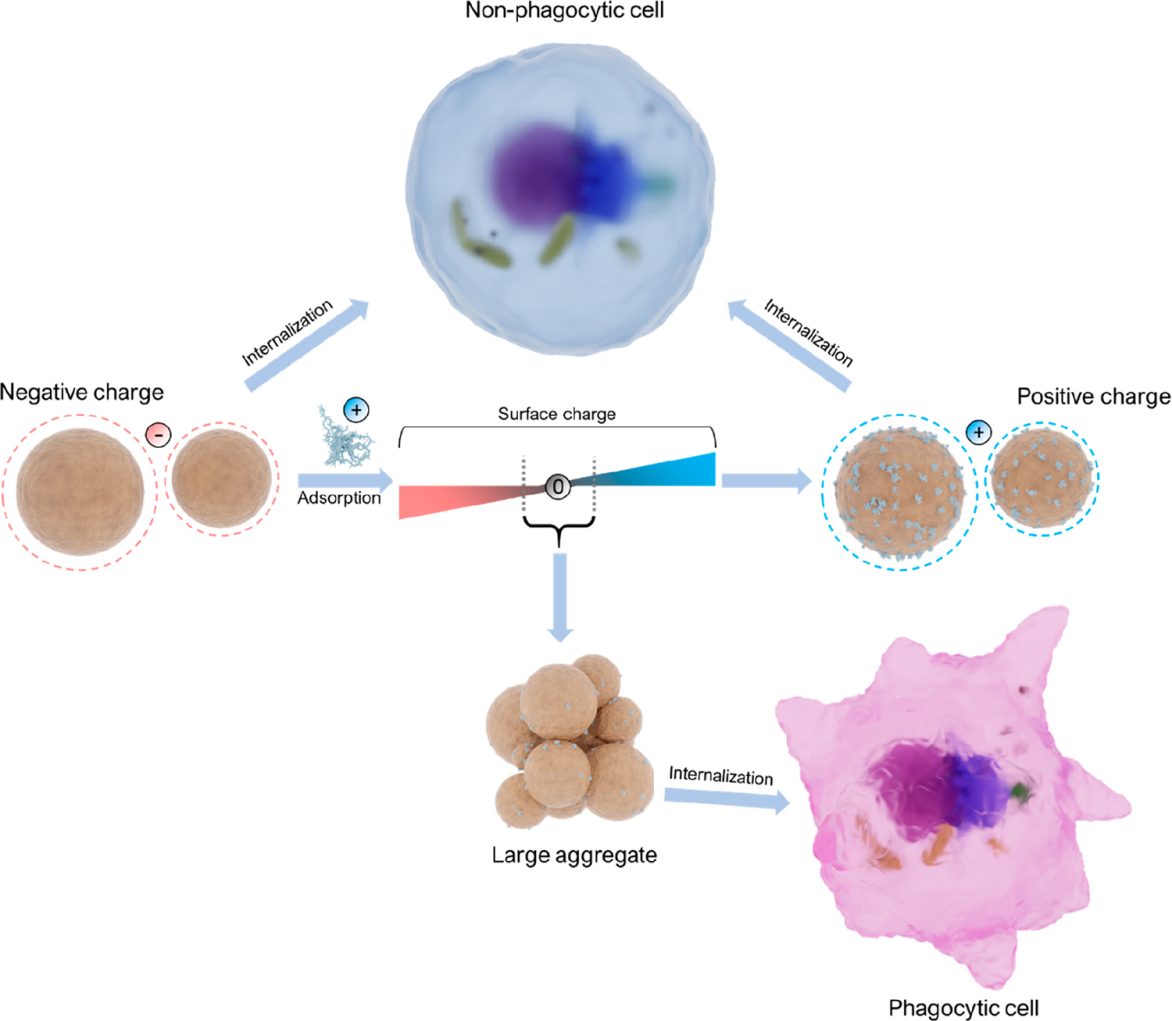
Aggregation and internalization specificity of nanoparticles toward nonphagocytic and phagocytic cells.

Surface chemistry also affects the internalization of NPs into cells. However, surface chemistry is difficult to control in vivo. For example, positively charged nanoparticles are often internalized more quickly than negatively charged particles,^313^ but positively charged nanoparticles are often eventually coated with various negatively charged proteins in vivo (Figure 14).^310^ The adsorption of serum proteins onto particles often leads to a decreased net charge, which can lead to aggregation via charge neutralization or bridging attraction.^217,294^ Aggregation eventually decreases specificity toward nonphagocytic cells. However, some serum substances can initiate clathrin-mediated endocytosis, and nanoparticles are therefore often coated with serum proteins, DNA polymers, saccharides, liposomes, or other substances that naturally occur within living organisms. On the other hand, some serum substances can also make it easier for phagocytic cells to detect and internalize nanoparticles,^309^ which is another reason to adsorb specific serum substances beforehand, thus preventing unwanted substances from adsorbing.

While multiple studies have demonstrated the adsorption of certain proteins onto LNPs for functionalization purposes,^314,315^ no study has done so with the aim of improving cell internalization in vivo. Figueiredo et al. nevertheless demonstrated the grafting of tumor-homing phoryn and iRGD peptides onto carboxylated LNPs to increase their internalization into certain kinds of cancerous cells in vitro.^298^ However, there is still a lack of investigations specifically exploring interaction mechanisms between LNPs and cells. Such studies would be valuable, as they would increase the understanding of important factors for the medicinal use of LNPs.

Toxicity has been briefly mentioned, but now we will move to discuss toxicity more specifically. The accumulation of particles in mononuclear phagocytic systems of central organs can lead to long-term toxic effects. One of the main tasks of the liver and kidneys is clearing foreign compounds and particles, but their ability to do so can vary.^316^ In an excellent study, Zelepukin et al.,^307^ comprehensively explored factors that affect the immune system’s ability to clear particles from the bloodstream. In addition to the factors that we have already discussed, they found that the nanoparticle-to-macrophage ratio was significant. Higher doses led to longer blood circulation times, seemingly because the mononuclear phagocyte system would get saturated. In fact, about 95% of all intravenously injected nanoparticles accumulate in the mononuclear phagocytic system’s organs, where they can create lesions and cause long-term inflammatory responses.^317^ It is unlikely that lignin can degrade in animal cells, which means LNPs could pose a problem in this regard. Particle accumulation of commonly used particles, such as silica, has led to the development of autoimmune diseases, chronic inflammation, cancer, and the production of reactive oxidative species that can spread beyond the accumulation site.^317^

Cytotoxic effects within cells can be caused in many ways. Once internalized, the cell will eventually attempt to metabolize the particles.^308,312,318^ Animal cells commonly use proteasomal or lysosomal pathways for protein degradation, but these mechanisms only work on proteins because certain amino acid structures are needed to initiate proteolysis.^319^ Lysosomal vesicles also drop the pH, which can help to degrade some persistent structures, but synthetic or inorganic nanomaterials, such as polystyrene, carbon nanotubes, gold, or silver particles can often resist these conditions.^320^ Nanomaterials that resist lysosomal action can lead to lysosomal dysfunction, commonly in the form of lysosome membrane permeabilization, which often leads to cell death. Importantly, it is unknown whether lignin resists lysosomal degradation or not.

Nevertheless animal cells deal with the accumulation of nondegradable protein structures frequently.^308,312,318^ For example, while mildly oxidized proteins can be metabolized through proteasomal or lysosomal pathways, heavily oxidized proteins can form large aggregates that resist proteolysis,^321^ such as oxidized low-density lipoproteins and lipofuscin, which is also formed by oxidation.^322^ Although the accumulation of both lipoproteins and lipofuscin is normal to some extent, it can lead to problems such as atherosclerosis and reduced lysosomal activity and dementia.^322,323^ Lignin nevertheless provides antioxidant properties, which could reduce the damage of reactive oxidative species, which promote the formation of nondegradable protein aggregates, lipofuscin, DNA damage, and more.^321,322,324^ Therefore, LNPs could be a good option to replace inorganic or synthetic particles for short and nonreoccurring treatments, such as cancer treatments, regardless of degradation. However, more studies on this topic are needed.

Nanoparticles are not only studied to be used as drug vectors in the biomedicinal field. Antibiotic-resistant bacteria are increasingly common globally, and nanoparticles have potential to be used as treatment when antibiotics fail.^325^ There have been a few studies on LNPs as antimicrobial agents, but many only use the LNPs as vector, e.g. for silver, which is the actual antimicrobial agent.^300,326,376^ However, LNPs have shown some antimicrobial properties against specific bacteria, but the mechanism behind the antimicrobial activity remains unknown.^300^ Still, if the surface chemistry and particle size are contributors to the antimicrobial activity, additional studies on other types of nanoparticles could provide insight into the mechanism for LNPs and how LNPs should be used best as an antimicrobial agent.

Lintinen et al. observed a rather high growth inhibition for unmodified LNPs against *Pseudumonas aeruginosa* and *Stapylococcus aureus* but not for *Escherichia coli*.^300^ Good growth inhibition against *S. aureus* but poor inhibition against *E. coli* was also observed by Gerbin et al.^327^ There is little knowledge available regarding the antibacterial mechanisms of nanoparticles against these bacteria, but it is unlikely that particles of above 100 nm are internalized through the rigid bacterial cell wall, so the disruption of the cell wall from the outside is most likely. In addition, because *P. aeruginosa* and *S. aureus* are particularly notorious biofilm producers, while *E. coli* both resides as individual cells and within biofilms,^328−334^ one reason for the LNP’s growth inhibition in these bacteria could be the inhibition of biofilm formation. For example, quercetin, a natural flavonoid with an aromatic structure, is able to inhibit biofilm formation in *P. aeruginosa* by adhering to the bacteria’s surfaces.^335^

Unmodified LNPs have shown low cytotoxicity against mammalian cells^199,266,297,298^ and could thus be suitable for tissue engineering.^199,299^ Nanoparticles have also gathered interest in tissue engineering as they can both be used to tailor the mechanical strength of scaffolds^199^ and work as vectors for bioactive agents.^336^ Although LNPs have not yet extensively been studied for tissue engineering, studies on their use as drug carriers and reinforcing materials in composites provide some foundational information. We thus discuss some ways in which LNPs could be used in this area.

Many scaffold matrices are at least partially fabricated from synthetic materials, while commonly used natural materials include collagen, actin, and fibronectin in addition to CNF discussed in section 3.4. Many synthetic polymers can elicit a foreign body response within the host cell upon plantation.^337,338^ Such a response can cause surrounding immune response cells to create a fibrotic capsule to seal off the foreign structure completely. Even synthetic materials that are widely used and regarded as prominent in tissue engineering can be rejected by the immune response.^338^ Lignin has not initiated such a response in various tests both in vitro and in vivo and could thus be a suitable biomaterial additive for scaffolds.^337^ In fact, lignin antioxidant properties have been beneficial to dampen harm to the surrounding cells and thus decrease rejection.^338^ Lignin nanofibers could be an interesting structural element in scaffolds and gels,^338−340^ and we thus refer to section 3.4 for a brief overview of the subject.

When the cells have differentiated in the scaffold, they start to produce their own ECM, which eventually replaces the scaffold’s matrix structure. The scaffold itself should therefore dissolve or diffuse over time, however, if the matrix does so too quickly, it may not provide enough time for cultured cells to create their own matrix structures or create disturbances in the surrounding tissue and initiate inflammation.^341^ Especially in load-bearing scaffolds (bones and articular cartilage), the strength and degradation speed are highly important because the combination of regenerated tissue and the scaffold should be able to bear sufficient load throughout the scaffold’s whole degradation process.^341−343^ Because the body has no mechanisms for degrading lignin, LNPs will likely not degrade in vivo, which makes it plausible that they could be used to prolong the scaffold’s breakdown time. Nanoparticles in general can be used to improve the mechanical properties and prolong breakdown times of scaffolds,^344−346^ and LNPs could likely be used for this as well.^199^

Cross-linking is another method used to increase a scaffold’s mechanical properties.^347,348^ LNP-reinforced scaffolds could likewise be strengthened by cross-linking LNPs with polymers in its matrix using calcium (electrostatic cross-linking)^349^ or epoxy chemistry (covalent cross-linking),^267,350,351^ for example. Because LNPs can provide strength both as a cross-linking site and as a particulate hardener,^199,250^ the balance between strength and degradation speed can be adjusted and tuned through particle size, concentration, and degree of cross-linking.

### 4.6 Lignin-Based Fibers

Although LNPs have dominated the discussion so far, we will also direct some attention toward lignin nanofibers, which is an emerging field. Because of its bulky and branched structure, lignin is a brittle material with a short effective “reach” despite its moderately high molecular weight. Lignin fibers and nanofibers therefore need linear copolymers as additives to increase intermolecular interactions and thus reduce brittleness. Various polymers can be used as copolymer and can be either blended in a melt with the lignin or grafted onto the lignin. However, because some lignins easily aggregate within polymer blends,^352^ copolymerization may be a better option depending on the lignin.^338,340,353^ While lignin-based fibers can be produced using a variety of methods, lignin nanofibers are primarily prepared using electrospinning.^354^ The thickness and strength of the fibers are affected by the force at which the fibers are pulled, that is, the voltage, the solution’s viscosity, and the solvent’s evaporation rate.^355,356^ The viscosity can be increased by increasing the concentration and especially by increasing the copolymer concentration, depending on its molecular weight. Therefore, spinning pure lignin fibers is highly challenging.^354^

The properties of the fibers depend on the lignin that is used, and fractionation can be used to obtain specific lignins to tailor the properties of the fibers. For example, electrospun fibers from acetone insoluble lignin and poly(ethylene oxide) had a higher heat storage moduli and better ability to retain their fiber morphologies when heated above 150 °C compared to fibers made from acetone-soluble lignin. In addition, acetone insoluble lignin produce hydrophilic fibers, while acetone soluble lignin fibers are rather hydrophobic.^353^ Hydrophilic fibers, such as those from acetone-insoluble lignin, may be useful in hydrogels or certain biomedical scaffold materials. Lignin’s antioxidant properties have been very useful in nanofibers for biomedical applications. For example, in section 4.5, we mentioned that many synthetic polymers can be problematic in biomedical scaffolds, as they lead to the formation of ROS. However, scaffolds from fibers of lignin-poly(lactic acid) copolymers and poly-l-lactide showed reduced formation of ROS and increased proliferation of stem cells.^338^ Similar results have been obtained for neurons and Schwann cells cultured on a substrate of nanofibers from lignin −polycaprolactone copolymers.^340^ The lignin fibers can also be surface modified. For example, although many lignin-based nanofibers are hydrophobic,^338,340,352^ hydrophilicity and interactions with salt ions can be increased by grafting poly-*N*-isopropylacrylamide brushes onto lignin-poly(ethylene oxide) fibers.^339^ Because the preparation of lignin-based nanofibers is versatile, and their properties can be modified according to application by choosing not only the lignin, but also the copolymer, lignin-based nanofibers have a lot of potential in various applications, including composites, biomedicine, energy storage, and aerospace.^338−340,352^ However, we note that more systematic research on the interactions between lignin and polymer in blends and between lignin and solvent during spinning would be beneficial for the optimization of these systems.

## 5 Lignin Containing Cellulose Nanomaterials

Recently, scientists have suggested combining the advantages of both cellulose and lignin in nanomaterials. There are different ways to achieve this. One approach is to produce CNMs with residual lignin, often called L-CNM in literature. L-CNF can be produced either by fibrillation of unbleached pulp^102,357−359^ or from biorefinery residues.^360^ When the lignin content is low (<20 wt %), the lignin generally facilitated fibrillation, while the opposite trend was observed for pulp with higher lignin content. Solala et al.,^357^ suggested that at low or moderate lignin content, the residual lignin is able to stabilize the free radicals that are formed during the mechanical grinding into stable phenoxy radicals. In the absence of lignin, the radicals were rapidly quenched. At high residual lignin content, the lignin is affecting the mechanical properties of the fibers, making them stiffer and restricting their swelling ability. As a consequence, the fibrillation efficiency is poorer. In an attempt to show full valorization of the lignin residues from a second-generation bioethanol production process, both LNPs and L-CNF was produced from the residue via acetone extraction. In this process, the majority of the polysaccharides had already been utilized for the bioethanol production and the residual fraction was partly degraded, hence the quality of the obtained L-CNF was not as high as achieved from unbleached pulp. Nevertheless surprisingly promising barrier properties were demonstrated for composite films including pure CNF, L-CNF, and LNPs.^360^

L-CNCs have also been produced to some extent, but the yield seems to generally be lower in the presence of lignin.^361^ Other methods to produce lignin-containing CNF are to adsorb soluble lignin,^362^ graft lignin to the CNF surface,^363^ or combine CNF and LNPs.^249,364^ These three approaches will result in slightly different surface properties. Unfortunately the literature on surface interactions of lignin containing CNMs is scarce. L-CNFs and L-CNCs have been observed to have slightly lower surface charge^365^ compared to pure CNMs. This could decrease their colloidal stability and make them more susceptible for aggregation by addition of salt. Water retention value (WRV) has been used to probe the interaction of L-CNF with water, and L-CNF has generally been found to have lower WRV than CNF from bleached pulp.^102^ However, we note that the water retention is related both to the degree of fibrillation as well as to the hydrophilicity of the fibril surface. Hence these two properties cannot be decoupled. Unbleached pulp may furthermore have a higher number of residual hemicelluloses. Residual hemicelluloses generally lead to increased swelling that consequently leads to both easier fibrillation and higher WRV^366^ adding to the challenge of addressing the surface chemistry of L-CNF only based on WRV. Consequently wetting studies using water contact angle measurements or other more surface sensitive methods like QCM-D should be applied to probe the effect of lignin on water interactions of CNMs.

The motivation behind using lignin-containing CNMs are 2-fold. On the one hand, the sustainability aspect of utilizing a waste stream for added value materials^360^ in the case of biorefinery residues or decreasing the processing steps when using unbleached pulp. On the other hand, all the anticipated benefits like compatibility with polymer matrix in composites, better water resistance, and resistance against oxidation and UV degradation are even more important motivations for using lignin containing CNMs. The use of CLPs together with CNF has the advantage of both nanomaterials being hydrophilic and easily dispersed in aqueous media. So far, these nanomaterials have been combined in films and hydrogels.^199,249^ However, more research is needed on the tuning of the surface chemistry of the LNPs for advanced materials as well as characterizing their interactions.

## 6 Techniques to Study Interactions

In the previous sections (sections 3.2–5), our current understanding of the surface properties and interactions of plant-based nanomaterials was reviewed. This understanding is based on the methods that have been available. To facilitate the choice of suitable methods for further investigations, some general information, advantages, and disadvantages of the main analytical methods used for the study of properties and interactions of biobased nanomaterials are summarized in Table 1.

**Table 1:** Analytical Methods Commonly Employed to Study Surface Properties and Interactions of Biobased Nanomaterials

Atomic force microscopy (AFM)	
Working principle	Sample preparation
For high-resolution images, the tip scans over the surface in a raster-scan manner, and the deflection of the cantilever is recorded with a laser beam reflected from the cantilever to a photodetector. Interaction forces are measured from the deflection of the cantilever while approaching and retracting the tip (or a colloidal probe) and the sample to/from each other.	No special sample preparation is needed for imaging.
	For force measurements, the molecules or materials of interest need to be immobilized on a substrate and on the tip or a colloidal probe attached to a tipless cantilever.
Accessible Info	
• Sample topography (3D nano/microscale images).	
• Mechanical properties (deformation, elastic modulus) of the sample surface.	
• Interaction and binding forces between molecules or materials.	
**Advantages**	**Limitations**
•3D images with subnanometric resolution.	• The samples to be imaged should not be too rough (typically roughness below a few micrometers).
• High sensitivity (∼ pN) for force detection within the range 10 pN to 100 nN.	• Information from only a small, local area.
• Both dry samples and samples in liquid can be analyzed.	• Vertical dimensions (height) are measured precisely, but lateral dimensions are usually overestimated because of the tip geometry (deconvolution corrections may be needed).
• Several scanning modes are available that can provide additional information on the mechanical and electrical properties of the sample.	• Sample preparation for force measurements can be time-consuming.
• It can follow changes in the sample in real-time.	• In force-vs-distance curves, the separation between the interacting surfaces or molecules is not directly measured but calculated by assuming that the samples are in contact at the maximum applied force on approach.
• Change of temperature or adsorption of molecules can be done in situ (e.g., to study the effect of polymer adsorption on the interaction between surfaces in liquid).	• The microscale roughness of the samples can affect the reproducibility of force measurements and their fitting with established theoretical models.
• Different setups are possible for force measurements (e.g., colloidal probe technique, single-molecule force spectroscopy, single-cell force spectroscopy).	• The attachment of cells to cantilevers for the measurement of cell-material interactions may compromise cell viability.

Scanning electron microscopy (SEM)	
Working principle	Sample preparation
A high-energy beam of electrons is focused onto a sample surface. The ejected X-rays, backscattered electrons, and secondary electrons are collected by the detectors and converted into a signal which forms the image.	Specific sample preparation is not required. However, to prevent charge buildup on the specimen surface, an ultrathin coating of gold, palladium, platinum, or other conductive material is needed (usually via sputtering).
Accessible Info	
• Shape and size of features in the specimen (high-resolution images at nano/microscale).	
**Advantages**	**Limitations**
• High-resolution images with nanometric resolution.	• Samples must be solid.
• Easy to operate and short measurement time.	• SEM cannot detect elements with atomic numbers <11.
• A variety of materials can be analyzed.	• Only surface features can be explored.
• Greater depth of focus and higher magnification compared to optical microscopy.	• The height of the features in the images is not accessible (no 3D information).

Quartz crystal microbalance with dissipation monitoring (QCM-D)	
Working principle	Sample preparation
Changes in the resonance frequency of a quartz crystal and the dissipation of the oscillation energy are measured during the adsorption or desorption of molecules or nanoparticles.	Adsorption can be measured directly on bare QCM-D sensors or model thin films deposited on the QCM-D sensors (e.g., by spin-coating). The analyte species is injected using a flow chamber.
Accessible Info	
• Adsorption and desorption kinetics.	
• Viscoelastic properties of the adsorbed layers.	
• Swelling and deswelling of thin layers.	
**Advantages**	**Limitations**
• High sensitivity in mass detection (≤1 ng/cm^2^).	• Model thin films are required as substrates.
• Adsorption can be monitored in real-time.	• Unable to distinguish selective adsorption of multiple component systems.
• It provides quantitative information on adsorbed mass and qualitative information on the viscoelasticity of the adsorbed layer.	• The sensed mass includes both substance adsorption and bound solvent molecules, and their respective contributions are difficult to decouple.
• Label-free method.	

Surface plasmon resonance (SPR)	
Working principle	Sample preparation
Plane-polarized light can generate surface plasmons at the interface between a metal (gold) and a dielectric medium at a particular angle (SPR angle) under total internal reflection conditions. The adsorption of molecules or nanoparticles causes a change in the refractive index leading to a change in the SPR angle, which is proportional to the mass adsorbed.	Adsorption can be measured directly on bare SPR sensors or model thin films deposited on the SPR sensors (e.g., by spin-coating). The analyte species is injected using a flow chamber.
Accessible Info	
• Adsorption and desorption kinetics.	
• Kinetics of intermolecular interactions.	
• Thickness and refractive index of the adsorbed layer when two wavelength lasers are used.	
**Advantages**	**Limitations**
• High sensitivity in mass detection (≤1 ng/cm^2^).	• Model thin films are required as substrates.
• Adsorption can be monitored in real-time.	• Unable to distinguish selective adsorption of multiple component systems.
• It provides quantitative information of adsorbed mass.	
• Label-free method.	
• Unlike QCM-D, SPR is an optical technique that is less sensitive to bound solvent molecules.	

Spectroscopic ellipsometry, flow cell (SE)	
Working principle	Sample preparation
Changes in the state of polarization of light are measured upon reflection at an interface.	No special sample preparation is needed.
Accessible Info	
• In situ adsorption.	• Interfacial mixing.
• Swelling and deswelling.	• Composition.
• Film thickness.	• Crystallinity.
• Refractive index.	• Anisotropy.
• Surface roughness.	• Uniformity.
**Advantages**	**Limitations**
• Fast and generally available.	• The standard analysis applies to only homogeneous, well-defined layers adsorbed on flat surfaces.
• Noninvasive, nondestructive.	
• Several film properties can be determined simultaneously.	• Difficult to set models for composite surfaces.

X-ray reflectivity (XRR)	
Working principle	Sample preparation
The sample surface is irradiated with a beam of X-rays over a range of angles close to the critical angle for total reflection. A portion of X-rays is reflected at every interface, creating a reflectometry pattern.	No special sample preparation is needed.
Accessible Info	
• The layer thickness of thin films and multilayers.	
• Surface and interface roughness.	
• Surface density gradients and layer density.	
**Advantages**	**Limitations**
• Fast measurements.	• Maximum film thickness ∼300 nm.
• Large areas can be analyzed (up to 300 mm).	• Surface roughness must be lower than ∼5 nm for thickness determination.
• No vacuum requirements.	
• Can be applied to conductive and insulating samples.	• Prior knowledge about the type of surface is required.
• In situ adsorption.	

Fourier-transform infrared spectroscopy (FTIR)	
Working principle	Sample preparation
The sample is exposed to IR radiation to excite vibrations in molecular bonds. The absorption peaks in the spectra at characteristic wavelengths indicate chemical groups present in the sample.	Samples in various states, e.g., solids and liquids, can be analyzed. No special sample preparation is needed.
Accessible Info	
• The molecular structure of the material.	
• Qualitative information on inter- and intramolecular interactions, water interactions, and conformational changes.	
**Advantages**	**Limitations**
• Nondestructive.	• Less surface-sensitive compared to XPS.
• Fast and accurate.	• Difficult to directly analyze the IR spectra qualitatively and quantitatively because bands are often wide, strong, and overlap with each other.
• Easy to operate.	• Solid samples should be in a dry form such as free-standing films or powders.
• Generally available.	• Water left in sample decreases accuracy.

X-ray photoelectron spectroscopy (XPS)	
Working principle	Sample preparation
The sample is irradiated with X-rays and emits photoelectrons. The intensity of the detected photoelectrons is then plotted as a function of their binding energy for analysis.	The measurements are done in (ultra)high vacuum conditions. Nevertheless, ambient pressure XPS tools also exist, so no vacuum is required. No special sample preparation is needed.
Accessible Info	
• Surface chemical composition and chemical bonds on the surface.	
**Advantages**	**Limitations**
• Surface sensitive.	• (Ultra)high vacuum is usually required.
• Quantitative chemical composition of the sample surface.	• The sample must tolerate (ultra)high vacuum.
• Relatively small sample amounts are needed.	• Only for surface analysis (analysis depth below 10 nm for classical Al Kα source).
	• Changes in sample surface due to water loss cannot be overruled.
	• Complex and expensive device.

Nuclear magnetic resonance (NMR)	
Working principle	Sample preparation
Constant and oscillating magnetic fields are applied to the sample and the response from the nuclei of the atoms is detected with sensitive radio frequency receivers.	In liquid-state NMR, samples with correct concentration are dissolved in suitable deuterated solvents and transferred to NMR tube (typically 5 mm diameter). Additional solvents generally need to be removed before sample preparation, solids need to be filtered, and in some cases, degassing is required.
	In solid-state NMR, samples are packed inside the rotors of various outer diameters (1–7 mm usually), usually made of zirconia.
Accessible Info	
• Provides a range of information: molecular structure, dynamics, interactions, physical parameters, and quantification.	
**Advantages**	**Limitations**
• Molecules can be measured in their native state.	• Expensive equipment and maintenance.
• Various nuclei can be detected.	• Spectral assignment and data analysis can be complex in some kinds of samples.
• Various 1, 2 and 3-dimensional experiments and their combinations can be utilized.	• Spectral interferences from impurities and solvent.
• Nondestructive technique.	• Not surface-sensitive.
• Amenable to many sample types: solutions, solids, tissues, and gas.	• Often requires the dissolution of samples.
• Spectral databases.	

Water contact angle (WCA)	
Working principle	Sample preparation
A drop of water is placed on the specimen surface, and the resulting angle formed by the droplet at the three-phase boundary (where liquid, gas and solid phases meet) is measured. For advancing and receding angles, the volume of the drop is increased or decreased, respectively, while measuring the contact angle in real time.	No special sample preparation is needed.
Accessible Info	
• Sample wettability (water contact angle).	
• Surface free energy, surface/interfacial tension.	
**Advantages**	**Limitations**
• Inexpensive.	• Artifact-prone, swelling of substrate.
• Simple and rapid.	• Measuring the WCA of highly hydrophilic surfaces is challenging.
• A small amount of water required.	• Surface chemistry information is not accessible.

Dynamic light scattering (DLS)	
Working principle	Sample preparation
The intensity of the light scattered by a dispersion fluctuates due to the Brownian motion of the particles. The analysis of those fluctuations provides the particle’s translational diffusion coefficient through which the hydrodynamic radius is obtained.	Dispersions should be prepared at an appropriate concentration, but otherwise, no special sample preparation is needed.
Accessible Info	
• Hydrodynamic radius.	
• Size distribution and polydispersity index (PDI).	
**Advantages**	**Limitations**
• Applicable to a wide range of materials.	• Particle geometry is ignored.
• Short measurement time.	• Overestimation of particle size due to hydration layer. Adjustment of sample concentration for optimal measurements.
• Noninvasive, simple.	
• Particle size range of 1–1000 nm.	

Table 1 describes methods that have been reasonably often used to study interactions of plant-based nanomaterials. Notably, the AFM in various configurations (colloidal probe microscopy, single cell force spectroscopy, or single molecule force spectroscopy) is one of the few methods that directly probe the force as a function of separation, enabling correlation to DLVO and other theories of colloidal stability. This has been successfully used for CNMs, but there are only a few studies on lignin model surfaces and no available reports on LNPs using force measurements. The various adsorption methods (QCM-D, XRR, SPR, and ellipsometry) have all been extensively applied especially for CNMs but lately also to a lesser extent for LNPs. QCM-D is probably the most commonly used method to probe adsorption, swelling, and deswelling kinetics as well as viscoelastic properties of layers. Due to the slightly different mechanisms of detection, a combination of methods is advantageous.^89,264,282^

The spectroscopic methods (NMR, FTIR, and XPS) are slightly different; they do not probe surface interactions in situ but can detect interaction like formation of new covalent or hydrogen bonds in bulk or, in the case of XPS, at the surface. Contact angle measurements are commonly applied to study the wetting properties of thin films from CNMs or LNM, that is, the interactions with water, while DLS can be used to probe aggregation of particles in dispersion but does not give very detailed information on interactions.

The analytical tools probing the surface interactions affecting nanomaterials assembly is rapidly evolving. Another positive trend is the increased efforts to apply computational and modeling tools to increase our understanding of the systems. Modeling and simulation can provide insight into the favorable mechanisms and thermodynamics during nanomaterial assemblies, interfacial dynamism between nanomaterial components, such as nanoparticle interactions with synthetic and biopolymers, polyelectrolytes, other nanoparticles and water along with molecular origin of interactions.^367^ It is not farfetched that deployment of these technological advances in experimental and numerical tools will lead to next-generation nanomaterials assemblies with tunable, adaptable, and interacting property pathways. Scattering methods have significantly increased our understanding of CNMs but have, with a few exceptions, not yet been applied to LNPs.^368−370^

With ever-expanding methods of nanoparticle synthesis and functionalization, a continuous improvement in highly accurate physical and chemical characterization methods is a prerequisite. Interestingly and notably, there are many characterization techniques which are utilized on a regular basis in the field of polymer science to gain fundamental understanding of polymer–polymer or polymer–nanoparticle interactions, but their adoption by the scientific community involved in the field of biobased nanomaterials has remained impeded. Such characterization techniques include mass spectrometry, isothermal titration calorimetry, sum frequency generation vibrational spectroscopy, and broadband dielectric spectroscopy. There are a few studies available indicating the potential of these methods.^131,371−373^ These are powerful analytical tools with the collective capabilities to study noncovalent interactions, analyze interactions by their thermodynamic patterns, molecular structures of buried solid/solid interfaces, and analyzing the molecular dynamics of polar segments that relax at the vicinity of nanoparticles. Without a doubt, the information gained from the above-mentioned tools is of unparallel importance, but it is likely that, due to the multidisciplinary and rapidly evolving nature of the bionanomaterials research field, a research group’s access to a range of characterization tools is limited. Furthermore, each characterization technique requires sophisticated data analysis and interpretation that can be beyond the expertise of a research group. Therefore it is imperative to build scientific collaborations to share knowledge, skills, tools, and techniques to accumulate a large body of knowledge that leads to scientific achievements and consequently economic development and growth.

## 7 Conclusions and Outlook

In this review, we have summarized the main surface characteristics of plant-based nanomaterials and their interactions with aqueous and nonpolar media as well as with polymers, proteins, and cells. We also described typical and less common methods that have been used for probing these interactions. Due to the extensive and thorough investigations of CNM interactions during the last 15 years, we have a reasonably good understanding of the main factors governing their behavior in aqueous media. Polymer adsorption to CNMs has also been rather extensively studied. Some key reports showing the effect of cellulose amphiphilicity on modification strategies for CNMs has advanced the use of CNMs in advanced materials. However, there are still knowledge gaps to be filled. One issue relates to the use of CNMs in biomedical applications, including tissue engineering and drug delivery. While there have been breakthroughs in, for example, the use of CNF hydrogels for wound healing, this research area would benefit from more understanding of the interactions between living cells and CNMs. The label-free and surface sensitive methods described in this review could nicely complement fluorescence microscopy and other assays more commonly used in biopharmaceutics. However, interdisciplinary collaboration is important to further development. Deep understanding of cell physiology is needed to ensure that cells stay alive during measurements and that relevant experimental setups are used, while fundamental understanding of the materials chemistry, colloid chemistry, and cell physiology is required for robust interpretation of results. Hydrogel stiffness and porosity is also essential for cell viability and transport of nutrients or drugs. While rheology measurements tell us about viscoelastic properties of the hydrogels, combining them with interactions studies using other methods would enable decoupling of the various reasons for changes in viscoelasticity like effect of solids content, charge water binding, particle size distribution, and aspect ratio. Differential scanning calorimetry thermoporometry measurements reveal pore size and volume in the nanometer range and could be used to a higher extent for hydrogel characterization. CNMs are also gaining interest in various foam or aerogel structures. More work on interaction with CNMs with nonpolar media could boost this research area.

Regarding LNPs, most focus has to date been on preparation and use of the particles, and significantly fewer efforts have been toward understanding their interactions. Compared to CNMs, this field is just emerging. A positive exception is the recent efforts combining detailed characterization with molecular modeling that has been applied to better understand the interactions governing the particle formation and parameters affecting the particle properties. However, due to the structural complexity of lignin, more research using different lignins, and looking more into the solvent–solvent interactions are desired for a better understanding of these complex supramolecular assemblies.

While it is indeed important to know how to tune the properties of LNPs, their surface interactions in applications are just as important but yet largely unexplored. This lack of knowledge is most probably hampering the efficient use of LNPs in applications. Herein, we note that because LNPs also bind water, similar effects due to water binding that have been observed for CNMs could occur also with LNPs. This phenomenon is worth further exploration, both fundamentally using surface sensitive techniques and in applications. We also note that, due to the complex chemistry of lignin and lack of fundamental studies, all kinds of interactions, ranging from hydrophobic, π–π and hydrogen bonds to electrostatic and vdW interactions are suggested when describing LNP interactions. However, these conclusions are seldom combined with experimental evidence. To date, direct interaction forces have not been measured using LNPs, but they could tell us what forces are dominating in specific situations. Furthermore, it is important to investigate how much variations in lignin source or particle preparation method affects interactions in final applications. This information would help in designing new materials; especially useful would be to probe how various surface modifications affect interactions. Not only AFM measurements but as large a variety as possible of different experimental methods in combination with molecular modeling should be applied to further our understanding of LNPs. This knowledge will play an important role in the development of any application for LNP. However, understanding and being able to tailor surface interactions is especially important for biomedical applications, where comprehending surface interactions is crucial to develop safe and effective treatments.

The need to combine methods does not only apply to LNPs but in general to all systems containing plant-based nanomaterials. Various methods probe interactions at different length scales (colloidal forces vs chemical bonds), in different states (dry or wet), or single interactions versus average over larger areas. Hence, one method is seldom enough. Just like scientists from other fields are getting interested in plant-based nanomaterials, we should look for potential new characterization methods more commonly used in other fields. The best methods depend on the intended applications. For example, in hydrogels, Pickering emulsions, and particle and film formation in aqueous media, the stiffness, rheological properties, self-assembly, stability, and distribution of components will be governed by long-ranged colloidal forces. As a consequence methods probing interactions in liquid, like colloidal probe microscopy, QCM-D, SPR, and calorimetry are useful. Then again short-ranged forces like hydrogen bonds and π–π interactions becomes important when discussing the strength of formed particles, films, and composites. To access these interactions, spectroscopy can be used but also other techniques that are less commonly used within the biomaterials research field should be explored. The importance of combining experimental studies with modeling and fundamental knowledge on interaction forces and polymer adsorption theory cannot be stressed enough to avoid misleading speculations and spreading of misconceptions. In summary, we hope that this review will inspire scientists to utilize the inherent surface properties of biobased nanomaterials to develop novel, value added, and sustainable materials.

## Abbreviations

AFM: atomic force microscopy
BNC: bacterial nanocellulose
Catlig: cationic lignin
C-PAM: cationic polyacrylamide
CLNP: carboxymethylated lignin nanoparticles
CLP: colloidal lignin particle
CMC: carboxymethyl cellulose
CMC-PEG: CMC with grafted PEG
CNC: cellulose nanocrystal
CNF: cellulose nanofibril
CNM: cellulose nanomaterial
DMA: dimethyl acetamide
DMSO: dimethyl sulfoxide
DSC: differential scanning calorimetry
DXN: 1,4-dioxane
*E. coli*: *Escherichia coli*
ECM: extracellular matrix
EDL: electrostatic double layer
EPTMAC: 2,3-epoxypropyl trimethylammonium chloride
FTIR: Fourier-transform infrared spectroscopy
G-lignin: guaiacyl lignin
GG: guar gum galactomannan
GGM: galactoglucomannan
H-lignin: *p*-hydroxyphenyl lignin
HepG2: human hepatocellular carcinoma cell line
L-CNC: lignin-containing CNCs
L-CNF: CNF containing residual lignin
L-CNM: CNM produced with residual lignin
LNP: lignin nanoparticle
LNM: lignin nanomaterials
NMR: nuclear magnetic resonance
*P. aeruginosa*: *Pseudumonas aeruginosa*
PAE: poly(amideamine) epichlorohydrin
PAH: poly(allylamine hydrochloride)
PCL: poly(ε-caprolactone)
PDADMAC: poly(diallyldimethylammonium chloride)
PEG: polyethylene glycol
PLA: poly(lactic acid)
PLNPs: carboxypentylated lignin nanoparticles
PVA: poly(vinyl alcohol)
PVAm: polyvinylamine
QCM-D: quartz crystal microbalance with dissipation monitoring
RH: relative humidity
S-lignin: syringyl lignin
*S. aureus*: *Stapylococcus aureus*
SMFS: single molecule force spectroscopy
SFA: surface force apparatus
SPAR: stagnation point adsorption reflectometry
SPR: surface plasmon resonance
TEMPO: 2,2,6,6-tetramethylpiperidine-1-oxyl
THF: tetrahydrofuran
TOCNF: TEMPO-oxidized cellulose nanofibers
vdW: van der Waals
WA07: human pluripotent stem cell
WRV: water retention value
XPS: X-ray photoelectron spectroscopy
XG: xyloglucan
